# Functional characterization of thioredoxin 3 (TRX-3), a *Caenorhabditis elegans* intestine-specific thioredoxin

**DOI:** 10.1016/j.freeradbiomed.2013.11.023

**Published:** 2014-03

**Authors:** María Jiménez-Hidalgo, Cyril Léopold Kurz, José Rafael Pedrajas, Francisco José Naranjo-Galindo, María González-Barrios, Juan Cabello, Alberto G. Sáez, Encarnación Lozano, Emma L. Button, Elizabeth A. Veal, Juan Carlos Fierro-González, Peter Swoboda, Antonio Miranda-Vizuete

**Affiliations:** a Centro Andaluz de Biología del Desarrollo, Departamento de Fisiología, Anatomía y Biología Celular, Universidad Pablo de Olavide, 41013 Sevilla, Spain; b Centre d’Immunologie de Marseille-Luminy, UM2 Aix-Marseille Université, Case 906, 13288 Marseille cedex 9, France; c Grupo de Bioquímica y Señalización Celular, Departamento de Biología Experimental, Universidad de Jaén, 23071 Jaén, Spain; d Center for Biomedical Research of La Rioja, 26006 Logroño, Spain; e Unidad Funcional de Investigación en Enfermedades Crónicas, Instituto de Salud Carlos III, 28220 Majadahonda, Madrid, Spain; f Institute for Cell and Molecular Biosciences, Newcastle University, Newcastle upon Tyne NE2 4HH, UK; g Center for Biosciences at Novum, Department of Biosciences and Nutrition, Karolinska Institute, S-14183 Huddinge, Sweden; h Instituto de Biomedicina de Sevilla, Hospital Universitario Virgen del Rocío/CSIC/Universidad de Sevilla, 41013 Sevilla, Spain

**Keywords:** *Caenorhabditis elegans*, Thioredoxin, Intestine, Stress, Pathogen infection, *Photorhabdus luminescens*, *Candida albicans*

## Abstract

Thioredoxins are a class of evolutionarily conserved proteins that have been demonstrated to play a key role in many cellular processes involving redox reactions. We report here the genetic and biochemical characterization of *Caenorhabditis elegans* TRX-3, the first metazoan thioredoxin with an intestine-specific expression pattern. By using green fluorescent protein reporters we have found that TRX-3 is expressed in both the cytoplasm and the nucleus of intestinal cells, with a prominent localization at the apical membrane. Although intestinal function, reproductive capacity, longevity, and resistance of *trx-3* loss-of-function mutants to many stresses are indistinguishable from those of wild-type animals, we have observed a slight reduction in size and a minor reduction in the defecation cycle timing of *trx-3* mutants. Interestingly, *trx-3* is induced upon infection by *Photorhabdus luminescens* and *Candida albicans,* and TRX-3 overexpression provides a modest protection against these pathogens. Together, our data indicate that TRX-3 function in the intestine is dispensable for *C. elegans* development but may be important to fight specific bacterial and fungal infections.

## Highlights

• *trx-3* encodes a novel member of the thioredoxin family in *Caenorhabditis elegans.*
• TRX-3 is the first metazoan thioredoxin specifically expressed in intestinal cells.
• TRX-3 is not required for intestine development or intestinal function.
• Specific bacterial and fungal infections robustly induce *trx-3* expression and TRX-3 overexpression moderately protects against infection.

The thioredoxin system is one of the most important systems for maintaining redox homeostasis in all eukaryotes [1]. Thioredoxins (TRXs) are a class of small multifunctional 12-kDa proteins that are characterized by the redox active-site sequence Trp-Cys-Gly-Pro-Cys (WCGPC) and a compact three-dimensional structure consisting of a central core of β-sheets surrounded by α-helices with the active site located in a protrusion of the protein [1]. In many cases, thioredoxin modules are found as part of multidomain proteins. Thioredoxins act as general protein disulfide reductases reducing many different substrates and becoming inactive in the process by the oxidation of the two cysteine residues at the active site. Oxidized thioredoxins are reactivated by thioredoxin reductases (TRXRs) at the expense of the reducing power of NADPH [2]. The *Caenorhabditis elegans* genome codes for several thioredoxin family members, although only a few of them have been characterized to date. TRX-1 is expressed in the bilateral sensory neuron ASJ and has been shown to regulate dauer formation and dietary restriction-mediated life-span extension [3–5]. TRX-2 is a mitochondrial thioredoxin of unknown function that is induced when the mitochondrial unfolded protein response is activated [6]. Both TRX-1 and TRX-2 have been shown to modulate the function of CEP-1 (the worm ortholog of the tumor suppressor p53) in neuronal integrity and life span [7]. A third member of the family is PNG-1, a peptide:*N*-glycanase that has an N-terminal thioredoxin domain and regulates axon branching [8,9]. Finally, DNJ-27 is the worm ortholog of human ERdj5, which has been recently shown to mediate some pathological phenotypes of *C. elegans* models of human neurodegenerative diseases [10].

We report here the genetic, cellular, and biochemical characterization of *C. elegans* TRX-3, the first example of a thioredoxin protein with an intestine-specific expression in eukaryotes. The intestine is one of the largest organs in the nematode *C. elegans* and its correct functioning is critical for many different physiological processes. For example, the intestine is responsible for food digestion, nutrient uptake, synthesis and storage of macromolecules, and elimination of the end products of metabolism [11]. Moreover, the posterior part of the intestine also acts as a pacemaker in the worm to regulate the defecation cycles [11]. Hence, a tight regulation of metabolite trafficking in the intestine is essential to control the energy metabolism of the organism [11]. Additional evidence implicates the intestine in the aging process. For instance, mutants of the *daf-16/FOXO* transcription factor have shorter life span, which is rescued to wild-type levels when DAF-16 activity is restored in the intestine [12]. Also, mutations in the intestinal peptide transporter *pept-1* have been shown to further extend the long life span of the insulin receptor *daf-2* mutants [13]. Being directly exposed to the environment, the intestine also constitutes a first line of defense against infection with pathogens that colonize this organ [14] and against toxicants and pollutants ingested with the food [11]. To cope with these stresses, the *C. elegans* intestine is equipped with a number of enzymatic defensive systems including heat-shock proteins, chaperones, and detoxifying and antioxidant proteins.

Using a *trx-3* loss-of-function mutant we demonstrate that TRX-3 is not essential for intestinal morphogenesis or for key cellular processes in which the role of the intestine is crucial, further supporting the notion that the thioredoxin system is dispensable for *C. elegans* development. However, we have uncovered a potential role of TRX-3 as protective system against some pathogen infections.

## Materials and methods

### C. elegans strains and culture conditions

The standard methods used for culturing and maintenance of *C. elegans* were as described previously [15]. The strains used in this work are described in Supplementary Table 1. All experiments were performed at 20 °C unless otherwise noted. All VZ strains were 6× backcrossed with N2 wild type.

### RNA extraction and RT-PCR analysis

For total RNA extraction, gravid hermaphrodites were washed off the plates with M9 buffer and dissolved in 5M NaOH bleaching solution. Embryos were collected and washed several times with M9 buffer. RNA was extracted from embryos using the NucleoSpin RNA II (Macherey-Nagel) kit following the manufacturer’s instructions. The total RNA was DNase-treated using Amplification Grade DNase I (Sigma), and 1 µg of DNase-treated RNA was reverse transcribed in a 20 μl reaction mixture. cDNA was generated using the iScript cDNA synthesis kit (Bio-Rad). One microgram of cDNA was used for RT-PCR using MBL-Taq DNA polymerase (Dominion-MBL) along with the corresponding primers for *trx-3* and *ama-1* genes (Supplementary Table 2) at a final concentration of 0.1 µM.

### Recombinant protein expression and purification

*trx-3* cDNA from the N2 wild type and the *trx-3(tm2820)* mutant was amplified with the forward primer 5′-CAGGGATCCGCTAAGAACTTTTTCTCCGG-3′ and the reverse primer 5′-GGCTGAATTCTTATTATGCACGGATTCTCTCG-3′ and cloned into the BamHI and EcoRI restriction sites of the pGEX-4T-1 vector to generate the constructs GST–CeTRX-3 and GST–CeΔTRX-3, respectively. These constructs were used to transform the *Escherichia coli* HMS174 strain, and recombinant protein expression was induced by adding 1 mM isopropyl-β-d-1-thiogalactopyranoside (IPTG) to a 100-ml LB medium bacteria culture of 0.5–0.7 OD supplemented with 0.1 mg/ml ampicillin and further incubating the cells at 25 °C and 200 rpm for 5 h. Cells were collected by centrifugation, immediately resuspended in 5 ml phosphate-buffered saline (PBS) containing 3 mg lysozyme, 0.5 mg DNase, and 0.5 mg RNase and incubated for 10 min at room temperature with gentle shaking. Next, the preparation was sonicated for 15 min on ice and the cell-free extract was obtained by centrifugation at 8000*g* for 30 min at 4 °C. Recombinant GST–CeTRX-3 and GST–CeΔTRX-3 proteins were purified from the cell-free extract using a Glutathione Sepharose 4B Affinity column (GE Healthcare) equilibrated with PBS and eluted with 50 mM Tris–HCl, pH 8, 10 mM glutathione. Finally, the purified protein was concentrated using Nanosep centrifugal devices equipped with a 10 K*-*MWCO Omega membrane (Pall Corp.).

### Thioredoxin activity assays

The enzymatic activity of the recombinant GST–CeTRX-3 and GST–CeΔTRX-3 proteins was tested by their ability to reduce bovine insulin A and B chains (Sigma, St. Louis, MO, USA) either using dithiothreitol (DTT) or NADPH (Sigma) and rat thioredoxin reductase-1 (IMCO, Stockholm, Sweden) as electron donors, as previously described [16], with slight modifications. Briefly, for the DTT assay, 25 μl of a reaction mix (composed of 40 μl of 1 M Tris–HCl, pH 7.5, 10 μl of 0.2 M EDTA, and 200 μl of 10 mg/ml bovine insulin) was mixed with the protein preparation in a final assay volume of 200 μl. The reaction was initiated by adding 2 μl of 100 mM DTT and the thioredoxin activity was measured by monitoring the increase in absorbance at 595 nm due to free insulin B-chain precipitation over time. For the NADPH and thioredoxin reductase assay, 20 μl of a reaction mix (composed of 40 μl of 1 M Hepes, pH 7.4, 8 μl of 0.2 M EDTA, 8 μl of 40 mg/ml NADPH, 100 μl of 10 mg/ml insulin) was mixed with the protein preparation in a final assay volume of 200 μl. The reaction was initiated by adding 1 μl of 1.5 mg/ml rat TrxR1 and the thioredoxin activity was measured by monitoring the decrease in absorbance at 340 nm due to NADPH consumption over time.

### Green fluorescent protein (GFP) and mCherry expression constructs, transgenesis, image capture, and analysis

A *trx-3* transcriptional GFP-fusion construct (*Ptrx-3::gfp*) was generated by amplification of the *trx-3* promoter region (2 kb) from N2 wild-type genomic DNA with the primers 5′-ACTCCTGCAGGGCCAATTTCATGATTTTCA-3′ and 5′-TCGAGGATCCAAAGTTCTTAGCCATTTCGA-3′ and cloned into the PstI and BamHI sites of the pPD95.77 vector. Likewise, a *trx-3* translational GFP-fusion construct (*Ptrx-3::trx-3::gfp*) was generated by amplification of the *trx-3* promoter plus genomic regions (4 kb) from N2 wild-type genomic DNA with the primers 5′-ACTCCTGCAGGGCCAATTTCATGATTTTCA-3′ and 5′-TGATGGATCCTGCACGGATTCTCTCGAGAT-3′ and cloned into the PstI and BamHI sites of the pPD95.77 vector. A *Ptrx-3::mCherry* transcriptional construct was generated by removing the *gfp* cassette with BamHI and EcoRI digestion from the above-mentioned *Ptrx-3::gfp* construct and replacing it with the *mCherry* cassette. To generate stable transgenic lines by microinjection, 10 ng/μl transcriptional *Ptrx-3::gfp* or *Ptrx-3::mCherry* construct and 50 ng/μl translational *Ptrx-3::trx-3::gfp* construct were injected along with either the *rol-6* (*su1006*) dominant transformation marker (50 ng/μl) or the *Punc-122::gfp* marker (50 ng/μl), respectively. To generate transgenic strains overexpressing wild-type TRX-3, the *trx-3* genomic sequence encompassing the *trx-3* 5′-UTR, genomic open reading frame (ORF), and 3′-UTR was amplified from the cosmid M01H9 with the primers 5′-ACTCCTGCAGGGCCAATTTCATGATTTTCA-3′ and 5′-GATCGGATCCTTTATATTTGATGTACATG-3′ and injected at 15 ng/μl along with the *Punc-122::gfp* marker (50 ng/μl). Several independent transgenic lines were isolated. For image analysis of fluorescent transgenic strains, worms were mounted in a 5 μl drop of 10 mM levamisole on a 3% agarose pad covered with a coverslip. Differential interference contrast (DIC) and fluorescence imaging was performed on a Zeiss AxioImager M2 with ApoTome unit fluorescence microscope. Images were captured with the AxioVision 4.8 software (Zeiss) and equal adjustment of brightness and contrast on control and matched problem images was implemented using Adobe Photoshop 10 software (Adobe Systems).

### RNA interference

The HT115 *E. coli* strain transformed with either pL4440 empty vector or the respective test clones was grown in liquid LB medium containing 100 μg/ml ampicillin for 15 h at 37 °C before being seeded on the RNAi plates containing 1 mM IPTG. The plates were incubated 2 days at 37 °C to induce dsRNA. Phenotypes were scored at 20 °C from the first generation onward by allowing interfered gravid hermaphrodites to lay eggs for 2 h on fresh RNAi plates.

### Developmental analyses

For the E blastomere cell lineage analysis, 4D microscopy was carried out using standard live-animal mounting techniques on a Leica DM6000 microscope fitted with DIC optics. The use of DIC optics allows cell tracing without using any dye or fluorescent marker that might alter the cell cycle progression. Embryonic cell lineage was determined as described [17]. In summary, gravid hermaphrodites were dissected and two- to four-cell-stage embryos were mounted on 4% agar pads in water and sealed with Vaseline. Imaging was performed at 25 °C. The multifocal time-lapse microscopy of the samples was controlled with the open-source software Micro-manager (www.micro-manager.org). Pictures on 30 focal planes (1 μm/section) were taken every 30 s for 12 h. Embryo lineages were analyzed with the software SimiBiocel [18]. For worm size determination, eggs from animals grown at 25 °C were harvested and allowed to hatch overnight in the absence of food. The synchronized larvae were transferred to NGM plates seeded with *E. coli* OP50 and kept for 48 h at 25 °C. Then, the animals on the plates were analyzed with the Union Biometrica COPAS automated sorter following the manufacturer’s instructions. The size of the worm (also known as time of flight) is generated in arbitrary, but constant, units and indicates the time required for the animal to travel through the measurement cell.

### trx-3 transcriptional regulation by TGF-β Sma/Mab

Synchronized late-L4 worms grown at room temperature were immobilized by transfer to unseeded NGM plates kept at 4 °C and imaged under a Leica stereomicroscope (M165FC) at 120× magnification. We estimated total mCherry expression of individual worms with ImageJ 1.46 c. Thus, we calculated the mean intensity value in the area of each measured worm and multiplied that mean by the area of each animal. At least 25 individuals were measured for every strain and experiment. Means and statistical and probability values from two independent experiments were obtained using Microsoft Excel and the program R version 2.15.1.

### Texas Red BSA and oil red O staining

To visualize apical intestinal endocytosis, L4 worms were soaked in a 0.1 mg/ml Texas Red BSA solution at 20 °C with shaking. After 4 h, the animals were washed twice with M9 buffer and placed on seeded NGM plates for 2 h to clean the intestine of residual marker [19]. To visualize intestinal fat accumulation, we used the neutral lipid dye oil red O as previously described [20]. Briefly, oil red O staining was conducted by PBS washing of 200–300 day-1 adult animals from plates initiated by synchronized egg-laying. To permeabilize the cuticle, worms were resuspended in 120 ml PBS to which an equal volume of 2× MRWB buffer (160 mM KCl, 40 mM NaCl, 14 mM Na_2_EGTA, Pipes, pH 7.4, 1 mM spermidine, 0.4 mM spermine, 30 mM 2% paraformaldehyde, 0.2% β-mercaptoethanol) was added. Worms were resuspended in 60% isopropanol, incubated 15 min at room temperature to dehydrate, and then stained overnight with 60% oil red O. Next, the residual dye was removed after allowing the worms to settle, and 200 ml of 1× PBS and 0.01% Triton X-100 was added. Image analysis was performed as described above.

### Stress assays

*Pgst-4::gfp* reporter induction in response to acrylamide treatment was performed as previously described [21] with slight modifications. L1-stage transgenic animals, synchronized by sodium hypochlorite treatment, were grown at 20 °C for 24 h on seeded NGM plates either containing or lacking 500 mg/L acrylamide. For the induction of the *Phsp-16.2::gfp* reporter upon heat-shock treatment, L4 transgenic animals grown on seeded NGM plates at 20 °C were incubated at 37 °C for 1 h, followed by a 2-h incubation at 20 °C, before the imaging analysis was performed. For the nuclear translocation of the *Pdaf-16::daf-16a::gfp* reporter, L4 transgenic animals, grown at 20 °C, were transferred to 37 °C for 30 min and visualized immediately afterward. For stress treatment survival analyses, animals not showing pharyngeal pumping or movement after mechanical stimulation were scored as dead and removed from the assay plates.

#### Juglone treatment

Thirty young adult gravid hermaphrodites were placed onto freshly made seeded NGM plates containing 240 µM juglone (Sigma) and viability was determined every 2 h during a total period of 8 h.

#### Paraquat treatment

One hundred L4 hermaphrodites were placed onto seeded NGM plates containing 4 mM paraquat (Sigma). Survival was monitored every day.

#### Heat-shock treatment

Thirty L4 hermaphrodites grown at 20 °C were placed on prewarmed seeded NGM plates and incubated at 37 °C. Survival was monitored every hour. The GraphPad Prism software package was used for graphical display and statistical analysis.

### trx-3 mRNA quantification

L4 worms were fed for 24 h at 25 °C with *E. coli* OP50, *Serratia marcescens* strain Db10, or *Photorhabdus luminescens* strain Hb. RNA isolation and cDNA generation were performed as described above. Quantitative real-time PCR was performed on a 7500 Fast Real-Time PCR system using 1 µl of cDNA in 10 µl of SYBR green (Applied Biosystems) and 0.1 µM corresponding specific primers (Supplementary Table 2). For *Candida albicans* experiments, young adult worms were fed with *E. coli* OP50 or *C. albicans* SN148 for 4 or 6 h. RNA extractions were carried out from triplicate samples using Trizol (Sigma) and *trx-3* and *act-1* mRNA levels determined using the Superscript III Platinum SYBR Green One-Step qRT-PCR kit (Invitrogen) and Corbett Life Science Rotor-Gene 6000 system. The results were normalized to *act-1* and then the relative expression was calculated. Control and experimental conditions were tested in the same run as technical triplicates. At least two independent experiments were performed.

### TRX-3 expression quantification

For TRX-3 expression quantification, L4 transgenic worms expressing the GFP reporter *Ptrx-3::trx-3::gfp* were grown on *E. coli, S. marcescens,* and *P. luminescens* at 25 °C. All micrographs were taken with identical image capture settings at 24 h postinfection and the quantification of GFP expression (measured as the fluorescence mean of 5–10 worms divided by the selected area and normalized by the background adjacent to the selected worm in the same image) was performed using the ImageJ software.

### Longevity assays

Life-span assays were performed at 25 °C as previously described [22], with slight modifications. Tightly synchronized embryos from bleached gravid adult hermaphrodites were allowed to develop through the L4 larval stage and then transferred to fresh NMG plates in groups of 25 worms per plate for a total of 100 individuals per experiment. The day the animals reached the L4 larval stage was used as *t* = 0. Nematodes not carrying the *fer-15 (b26)* mutation were transferred to fresh plates daily until progeny production ceased and after that were transferred every second to third day but monitored daily for dead animals. Nematodes that did not respond to gentle prodding and displayed no pharyngeal pumping were scored as dead. Animals that crawled off the plate or died due to internal hatching or extruded gonad were censored and incorporated as such into the data set. All longevity assays were repeated two times.

### Dauer formation assay

Analysis of dauer formation was performed as previously described [23]. Briefly, hypochlorite-purified eggs of each worm strain were spotted onto 60-mm plates and incubated at 20 and 25 °C. OP50 bacteria were not spread to the edges of the plates in order to minimize the number of dauer larvae crawling off the plate. Dauer and nondauer larvae were then counted as the first nondauers reached egg-laying age.

### Pathogen killing assays

For *Pseudomonas aeruginosa* PA14, killing assays were performed as previously described [24], with slight modifications. We plated 10 μl of a saturated culture of *P. aeruginosa* PA14 in LB-modified NGM plates and incubated for 24 h at 37 °C followed by another incubation at 25 °C for 24 h. Killing assays were carried out in triplicate and performed by manually transferring L4-staged animals from *E. coli* OP50 plates to pathogen plates. The killing plates were incubated at 25 °C and worm mortality was monitored at various points along the time course of death. For *S. marcescens* strain Db10 and *P. luminescens* strain Hb, killing assays were performed as described [25] and repeated three times. For *C. albicans* strain SN148 killing assays, young adult *C. elegans* were transferred from *E. coli* OP50 plates onto unseeded brain heart infusion (BHI) plates, then onto BHI plates containing 7.7 µM kanamycin and 100 µM FUDR seeded with a lawn of *C. albicans. C. elegans* survival was monitored at 25 °C. We used the GraphPad Prism software package for graphical display and statistical analysis.

## Results

### C. elegans TRX-3 is a functional thioredoxin specifically expressed in the intestine

**Fig. 1:**
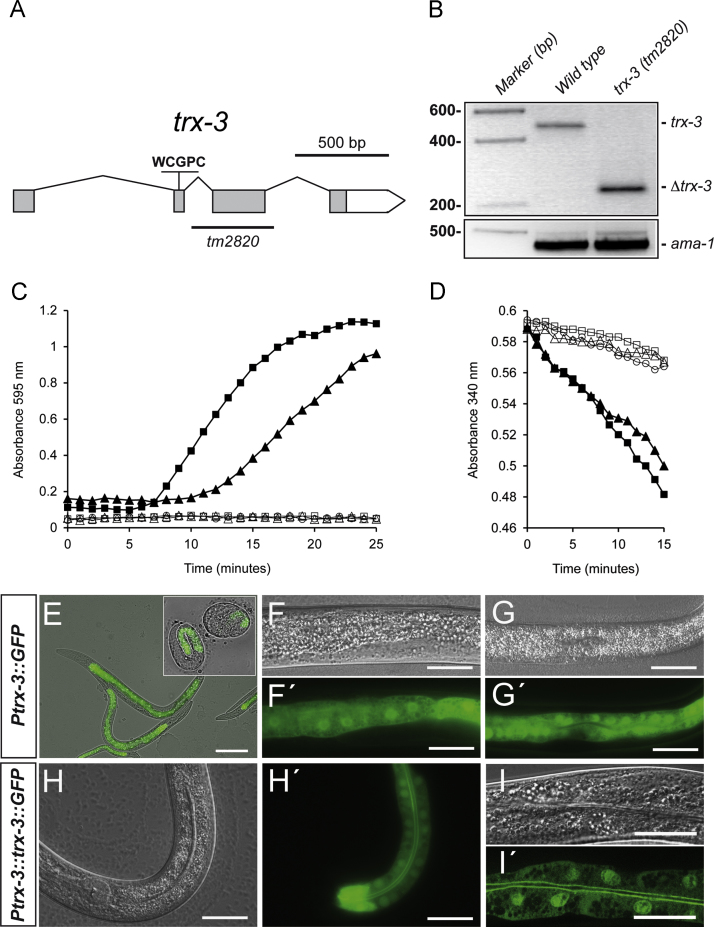
(A) Exon–intron structure of the *trx-3* gene. Boxes represent exons and lines introns. The gray boxes indicate the ORF and the white box indicates the 3′-UTR. The sequence and position of the redox active site are shown and the line underneath denotes the region deleted in the *tm2820* allele. (B) cDNA expression of the *trx-3* gene from N2 wild type and the *trx-3(tm2820)* mutant as determined by RT-PCR. The *ama-1* gene was used as a housekeeping gene loading control. (C, D) Enzymatic activity of TRX-3 and ΔTRX-3. Recombinant *C. elegans* GST-tagged TRX-3 (GST–CeTRX-3) and a truncated variant from the conceptual translation of the *tm2820* allele (GST–CeΔTRX-3) were assayed for their ability to reduce insulin disulfide bonds using (C) DTT or (D) NADPH and rat thioredoxin reductase-1 as electron donor [16]. (▪) GST–hTRX-1; (♦) GST–CeTRX-3; (□) GST–CeΔTRX-3; (▵) GST–CeTRX-3 without DTT (in C) or thioredoxin reductase (in D); (○) reaction mix only. Increase in absorbance at OD_595_ measures insulin precipitation upon reduction and decrease in absorbance at OD_340_ measures NADPH consumption. Recombinant human GST-tagged TRX-1 (GST–hTRX-1) was used as positive control. One representative experiment of two with similar results for each condition is shown. (E–I) Expression pattern of *trx-3* in the intestine. Transgenic worms carrying different expression constructs were visualized using (F–I) DIC or (F′–I′) fluorescence optics, except for (E), in which the composite image is shown. A transcriptional *Ptrx-3::gfp* fusion shows fluorescence in intestinal cells from embryo (inset in (E)) to adult with both nuclear and cytoplasmic signal (E–G). A translational *Ptrx-3::trx-3::gfp* fusion shows a prominent signal at the apical intestinal membrane and in some intestinal nuclei, whereas the cytoplasmic signal is substantially lower (H–I). Scale bar, 20 μm.

The *C. elegans* genome encodes nine members of the thioredoxin system with the conserved active-site sequence WCGPC (Supplementary Table 3), of which only TRX-1, TRX-2, PNG-1, and DNJ-27 have been characterized to date [3,6,8,10]. To gain further insight into novel members of the thioredoxin family we aimed to characterize the function of the worm TRX-3 protein, based on its homology to the previously characterized human and *C. elegans* thioredoxins (Supplementary Fig. 1A). The *trx-3* gene is composed of four exons and spans about 1.75 kb on linkage group IV (www.wormbase.org, version WS236). The *tm2820* allele is a 340-bp deletion that completely removes the third exon (Fig. 1A), and an RT-PCR analysis identified a truncated mRNA species in extracts from the *tm2820* mutant (Fig. 1B). The conceptual translation of the *tm2820* mRNA variant produces an aberrant protein (ΔTRX-3) that lacks amino acid residues 51 to 142, which correspond to those composing exon 3 (Supplementary Fig. 1B), whose absence is predicted to dramatically alter the three-dimensional structure of the native TRX-3 protein (Supplementary Fig. 1C). Consistent with this prediction, recombinant ΔTRX-3 is devoid of enzymatic activity in both the DTT and the NADPH/thioredoxin reductase assays, whereas native TRX-3 is fully active (Figs. 1C and D). Therefore, we conclude that *trx-3(tm2820)* is a loss-of-function allele.

Next, to identify the cells and tissues in which the gene *trx-3* is expressed, we generated transgenic animals expressing transcriptional (promoter only) and translational (promoter plus gene) GFP fusions. The transcriptional construct *Ptrx-3::gfp* was expressed exclusively in the worm intestinal cells starting in the embryo at the gastrula stage (the time at which the intestine specification occurs) and continued to be expressed in a gut-specific manner in the subsequent embryonic development, larval, and adult stages (Figs. 1E–G). Interestingly, the translational construct, *Ptrx-3::trx-3::gfp,* showed intense fluorescence at the apical (luminal) membrane of the intestine and the nucleus, whereas the cytoplasmic labeling was weaker (Figs. 1H–I).

### trx-3 is dispensable for intestine integrity and function

**Fig. 2:**
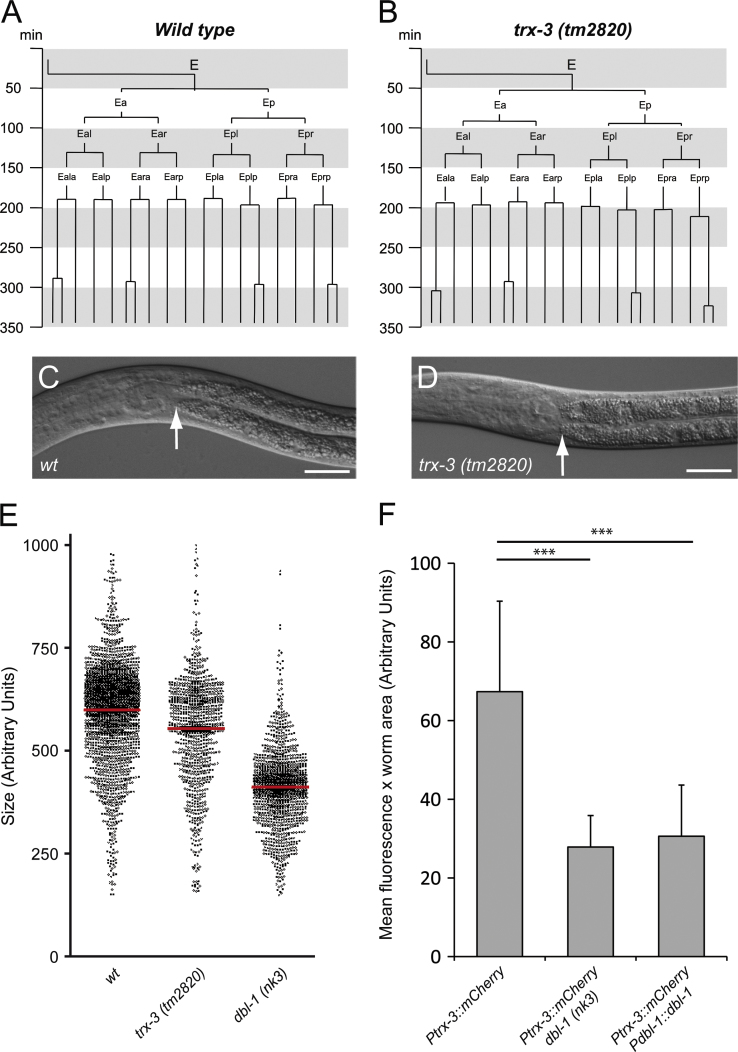
(A, B) The E blastomere cell lineage in wild-type and *trx-3(tm2820)* animals. Timing, in minutes, of cell cleavages derived from the E blastomere is shown. The invariant pattern of cleavages for wild type and one example of the very similar pattern of the *trx-3(tm2820)* mutant are given. There are no further cell divisions in the wild-type E lineage. We analyzed the cell lineage up to ~350 min of embryonic development; any cleavages beyond this time would not be detected because of the embryo movement. (C, D) The intestine of *trx-3(tm2820)* mutants has normal gross morphology as visualized by DIC optics. The arrow indicates the pharyngeal–intestinal valve position. Scale bar, 20 μm. (E) Size measurements of individuals from age-synchronized populations using the Union Biometrica COPAS automated sorter. Each dot represents one animal, the red bar is the average, and the size is in arbitrary, but constant, units. One single experiment is shown of three with similar results. (F) Quantification of mCherry expression driven by the *trx-3* promoter in wild-type, *dbl-1*-null mutant, and *dbl-1*-overexpressing backgrounds. Data represent two independent experiments with at least 25 animals per assay. Error bars indicate SEM. Two-tailed Student *t* test was used to determine statistical significance (^⁎⁎⁎^*p* < 0.001).

The restricted expression pattern of the *trx-3* gene in gut cells prompted us to investigate whether *trx-3* is required for intestinal function. *trx-3* RNAi interference in both wild-type and *rrf-3(pk1426)* hypersensitive backgrounds did not produce any phenotype (data not shown). Consistent with this result, *trx-3(tm2820)* mutants did not show any obvious developmental (Figs. 2A and B) or morphological defect in the gut throughout their life cycle (Figs. 2C and D). However, subtle phenotypes were identified in *trx-3(tm2820)* animals, such as a smaller size (Fig. 2E) and a shorter defecation cycle (Table 1). One of the pathways that regulates body growth in *C. elegans* is the TGF-β Sma/Mab pathway. Worms unable to synthesize the ligand DBL-1 or any of the downstream components of the pathway are dwarf. However, animals that overexpress DBL-1 become long. Moreover, DBL-1 has also been shown to regulate the development of male tail structures and worm innate immunity [26,27]. Thus, we asked whether the small size of *trx-3(tm2820)* mutants could be caused by regulation of *trx-3* expression by TGF-β Sma/Mab signaling. For that purpose, we crossed the *Ptrx-3::mCherry* reporter strain with a *dbl-1-*null mutant and a *dbl-1*-overexpressing strain. As shown in Fig. 2F, both genetic backgrounds cause a downregulation of the *Ptrx-3::mCherry* reporter, suggesting that proper DBL-1 levels are needed to keep a regular *trx-3* expression.

Importantly, the absence of any developmental or morphological defect in *trx-3(tm2820)* mutants is not a consequence of redundancy with either of the two other described worm thioredoxins, *trx-1* and *trx-2* [3,6]. Hence, double mutants *trx-1(ok1449); trx-3(tm2820)* and *trx-2(tm2720); trx-3(tm2820)* are viable, do not display any obvious developmental or morphological phenotype, and have a life span comparable to that of the single *trx-1* or *trx-2* mutants (Supplementary Fig. 2). Furthermore, we failed to identify any developmental or morphological synthetic interaction of *trx-3(tm2820)* mutants when treating them with RNAi targeting all known members of the thioredoxin family and closely related glutaredoxin and peroxiredoxin protein family members in *C. elegans* (Supplementary Table 4), making it unlikely that *trx-3* is functionally redundant with one of these genes. However, it remains possible that RNAi did not completely ablate the expression of these genes or that several of these genes may be functionally redundant with *trx-3*.

**Fig. 3:**
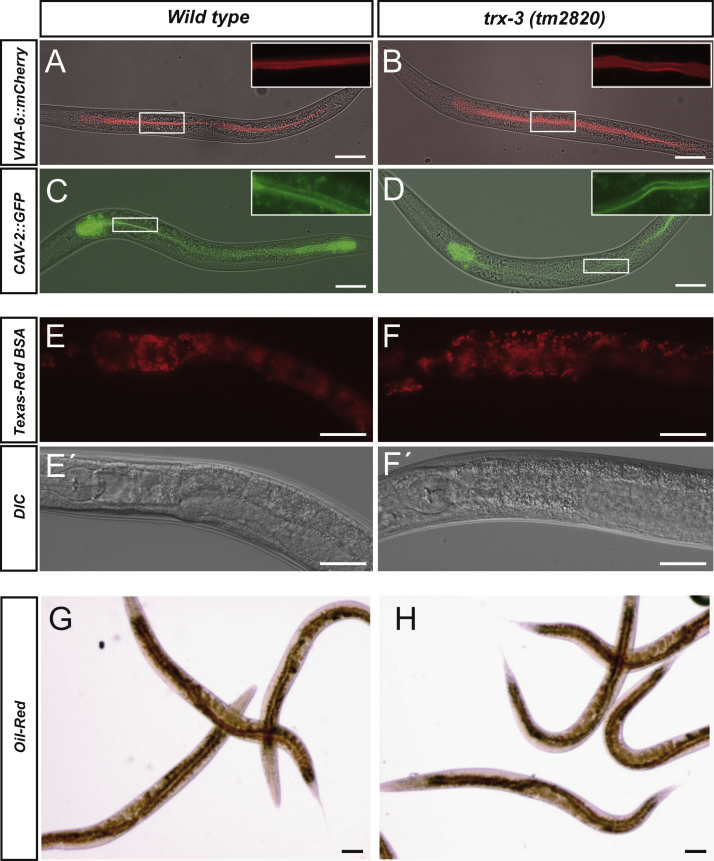
(A–D) The intestinal apical membrane maintains its structural integrity in *trx-3(tm2820)* mutants as demonstrated by the normal fluorescence distribution of VHA-6::mCherry and CAV-2::GFP markers [28,29]. Images are composites of fluorescence and DIC optics. Insets show the magnification of the boxed area in the fluorescent channel. (E, F) Luminal incorporation of the fluorescent dye Texas Red BSA. Both wild-type and *trx-3(tm2820)* worms show similar levels of gut red fluorescence after 24 h incubation with 0.1 mg/ml dye. (E′, F′) DIC optics of the same worms. (G, H) Intestinal fat storage with both wild-type and *trx-3(tm2820)* worms displaying comparable amounts of lipids in the intestine as determined by the neutral lipid dye oil red O staining [20]. Scale bar, 20 μm.

Given the prominent expression of TRX-3 in the apical intestinal membrane, we next asked whether TRX-3 function is required to maintain its structural integrity or the proper localization of other apical markers. As shown in Figs. 3A–D, the continuity of the apical membrane is maintained in a *trx-3(tm2820)* mutant background, demonstrated by the normal apical distribution of well-known apical markers such as VHA-6, a vacuolar ATPase [28], or CAV-2, a member of the caveolin family [29]. Interestingly, caveolins have been shown to interact with the thioredoxin system in mammals [30]. Given that both TRX-3 and CAV-2 are located at the intestinal apical membrane, we asked whether *trx-3* and *cav-2* mutants display any synthetic genetic interaction. However, *trx-3(tm2820); cav-2(tm394)* double mutants had no obvious phenotype except for the slight brood-size reduction already reported for the *cav-2(tm394)* single mutant (data not shown) [29]. An important function of the intestine is the uptake of nutrients [11] and, because of the apical localization of TRX-3, we asked whether the luminal content uptake was compromised in *trx-3(tm2820)* animals. To this purpose, we fed worms with the fluorescent marker Texas Red BSA [29] and demonstrated that *trx-3(tm2820)* mutants incorporate the luminal marker at a rate similar to that of the wild-type control (Figs. 3E and F), suggesting that TRX-3 is not essential for luminal endocytosis and that the apical membrane functionality is not compromised in these mutants. Consistently, fat content and distribution in *trx-3(tm2820)* mutants were comparable to those of wild-type control worms (Figs. 3G and H). We therefore conclude that TRX-3 is not essential for intestinal function under normal growth conditions.

### trx-3 does not play a protective role against chemical or heat stress in C. elegans

**Fig. 4:**
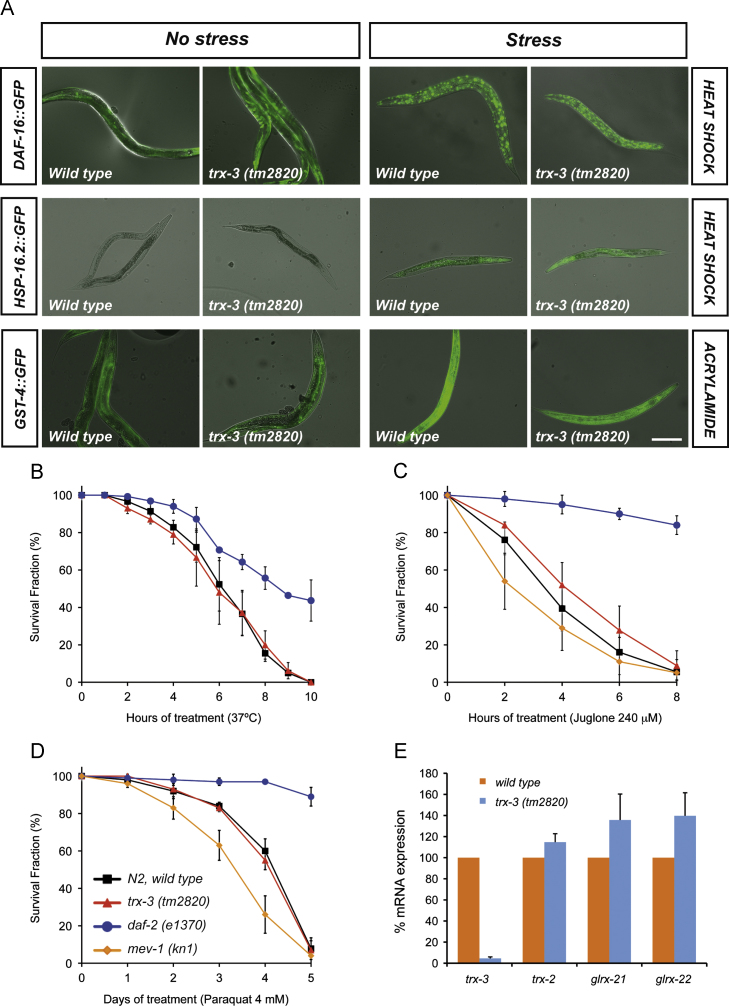
(A) Transgenic worms expressing the fluorescent stress markers DAF-16::GFP, HSP-16.2::GFP, and GST-4::GFP in wild-type and *trx-3(tm2820)* mutant backgrounds show comparable levels of activation upon heat stress and acrylamide treatment. Images are composites of fluorescence and DIC optics. Scale bar, 200 μm. (B–D) N2 wild type and *trx-3(tm2820)* mutants were assayed for their resistance to various stress treatments such as (B) heat shock at 37 °C, (C) juglone at 240 μM, and (D) paraquat at 4 mM. Graphs represent the average of three independent experiments. All treatments (except heat shock) were carried out at 20 °C. Error bars indicate the standard error of the mean. *daf-2(e1370)* and *mev-1(kn1)* mutants were used as resistant and sensitive controls, respectively. Differences between N2 wild-type and *trx-3(tm2820)* animals were not significant in all cases by one-way ANOVA (*p* > 0.05). (E) Quantification of *trx-2, trx-3, glrx-21,* and *glrx-22* mRNA levels by qPCR of N2 wild-type animals and *trx-3(tm2820)* grown on *E. coli* OP50. Bars represent the percentage of each mRNA species±SEM in the two different genetic backgrounds calculated from three independent assays. Differences were nonsignificant in all cases by two-tailed Student *t* test (*p >* 0.05).

The *C. elegans* intestine is constantly exposed to chemical stressors and toxins as well as pathogens [11], and thioredoxins are well-known antioxidant proteins that have been shown to function as protective systems against various types of stresses [31]. Thus, we decided to test whether TRX-3 plays a role as an anti-stress defense mechanism in the *C. elegans* intestine. With this objective, we first studied whether *trx-3(tm2820)* mutants modified the induction or subcellular distribution patterns of well-known GFP markers of stress such as DAF-16, HSP-16.2, or GST-4. DAF-16 is a FOXO-type transcription factor that normally resides, inactive, in the cytoplasm but translocates into the nucleus upon stress, where it mediates the transcription of many genes involved in stress defense [32]. Importantly, DAF-16 function in intestine has been linked to fat storage and longevity [12]. HSP-16.2 is a small heat-shock protein, direct target of DAF-16, that is activated upon thermal and oxidative stress [33]. GST-4 is a glutathione *S*-transferase that has been shown to be induced upon exposure to diverse toxic chemicals such as acrylamide or methylmercury [21,34]. As shown in Fig. 4A, heat shock or acrylamide treatment did not alter the nuclear translocation of DAF-16 or induction of HSP-16.2 and GST-4 GFP reporters in *trx-3(tm2820)* animals compared with the age-matched wild-type controls. In addition, *trx-3(tm2820)* mutants subjected to heat shock and chemically induced oxidative stress by paraquat and juglone did not show enhanced lethality either (Figs. 4B–D). As shown in Fig. 4E, the expression levels of *trx-2, glrx-21,* and *glrx-22* (the members of the thioredoxin/glutaredoxin family with known expression in gut; [6] and A. Miranda-Vizuete, unpublished observations) were not changed significantly in a *trx-3(tm2820)* mutant background. Consistent with these results, the redox status of *trx-3(tm2820)* mutants is not significantly different from that of wild-type controls, as determined by the levels of oxidized versus reduced PRDX-2 [35,36] or by using the genetically encoded fluorescent biosensor HyPer [37] (Supplementary Fig. 3). As a whole, it seems unlikely that this lack of enhanced sensitivity to stress associated with loss of *trx-3* is due to a compensatory mechanism involving other closely related stress-response genes. Instead, our data indicate that TRX-3 does not play a key role in the general anti-stress response in *C. elegans*.

### daf-2-dependent phenotypes are not regulated by trx-3

**Fig. 5:**
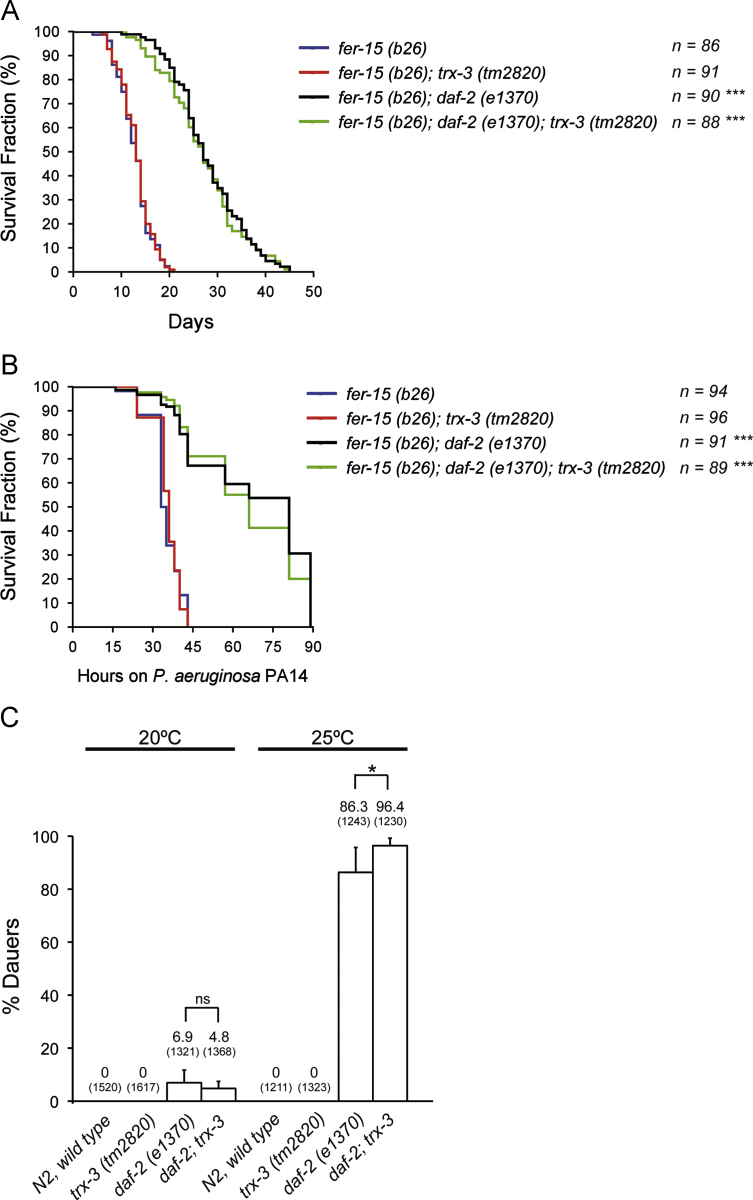
(A) The longevity of the *trx-3(tm2820)* mutant on *E. coli* OP50 and (B) survival after *P. aeruginosa PA14* infection were assayed at 25 °C in *fer-15(b26)* and *fer-15(b26); daf-2(e1370)* backgrounds. The *fer-15(b26)* mutation was included to prevent internal hatching of the *daf-2(e1370)* progeny and has been shown not to influence longevity [56]. Kaplan–Meier plots were used to show the fraction of animals that survived over time. Longevity and infection survival assays were performed twice, obtaining similar results, and the composite data are shown. The survival rate of *trx-3(tm2820)* animals was compared to that of their respective controls, wild type for *trx-3,* using the log-rank (Mantel–Cox) test, and the differences were not significant in all cases (*p* > 0.05). When comparing *daf-2* backgrounds versus their corresponding non-*daf-2* controls, the differences were highly significant (^⁎⁎⁎^*p* < 0.001). (C) Effect of the *trx-3(tm2820)* allele on the formation of dauers by *daf-2(e1370)* animals. Assays were performed at 20 and 25 °C on 10 independent plates initiated with 100 to 150 eggs per plate. Numbers above the bars show the percentage of dauers and the numbers in parentheses indicate the total number of animals scored. Error bars indicate SD. χ^2^ test was used to determine statistical significance (ns, not significant; ^⁎^*p* < 0.05).

Compromised nutrient uptake has been associated with increased life span. This is illustrated by the extended longevity of *eat-2* mutants that have reduced food intake due to decreased pharyngeal pumping [38]. In this context, reduced function of *nhx-2,* a nutrient transporter located in the intestinal apical membrane, promotes life-span extension [39]. Moreover, the longer life span of *daf-2* insulin receptor mutants is further enhanced by mutations in *pept-1,* another peptide transporter [13]. This last study also showed that *trx-3* mRNA is induced 5.65-fold in *daf-2* single mutants and 7.56-fold in a *pept-1; daf-2* double-mutant background (B. Spanier, personal communication). For this reason, we decided to study if *trx-3* had any impact on *daf-2*-dependent traits such as longevity, dauer formation, or survival against *P. aeruginosa* PA14 infection [40,41]. Surprisingly, despite the remarkable induction of *trx-3* mRNA in a *daf-2* background, *trx-3(tm2820)* mutants did not show any effect on any of these traits, either alone or in combination with a *daf-2(e1370)* mutation (Fig. 5).

### C. elegans trx-3 is specifically induced upon P. luminescens and C. albicans infection but it is not essential for pathogen infection survival

**Fig. 6:**
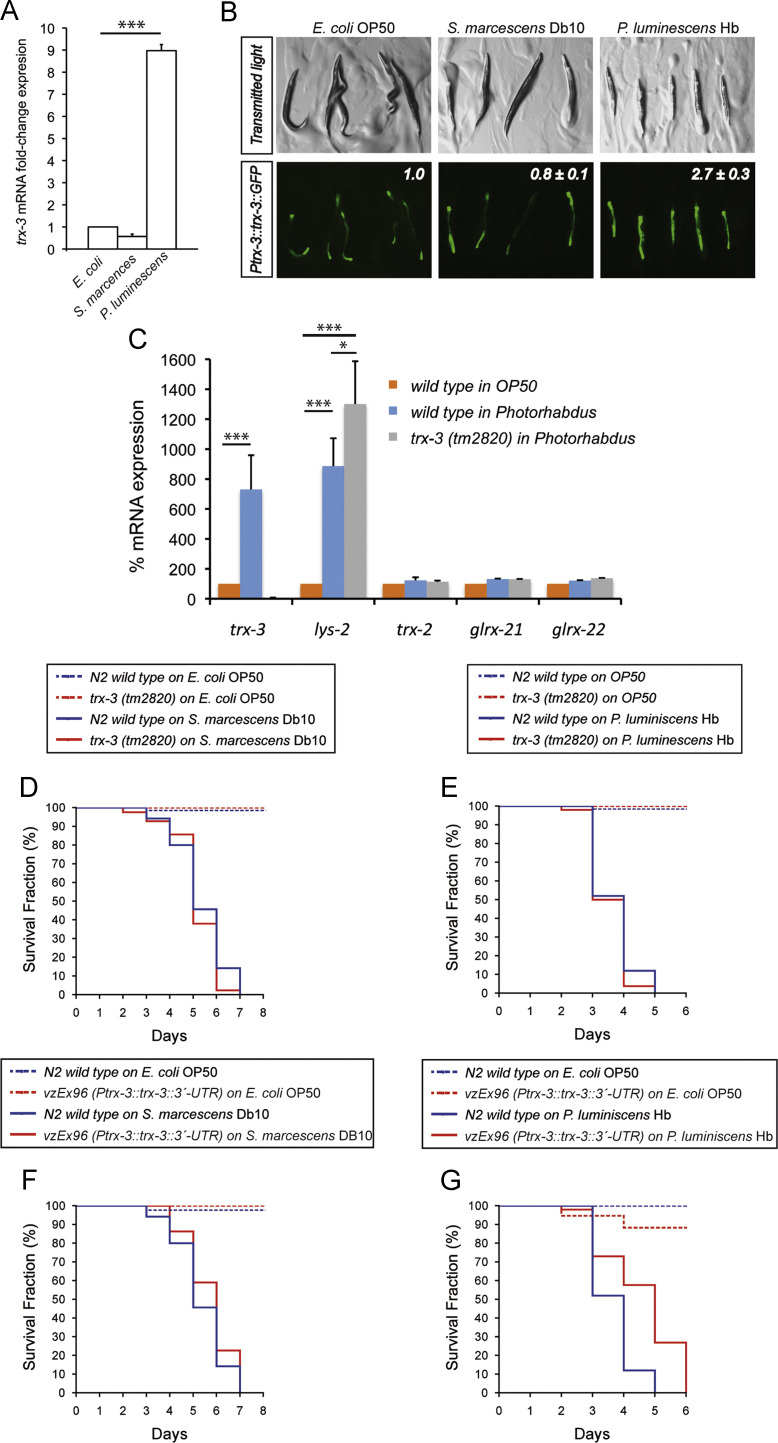
(A) Quantification of *trx-3* mRNA levels by qPCR of N2 wild-type animals grown on *E. coli* OP50, *S. marcescens* Db10, and *P. luminescens* Hb. Bars represent the average fold change in expression of three independent assays±SEM. Two-tailed Student *t* test was used to determine statistical significance (^⁎⁎⁎^*p* < 0.001). (B) Transgenic worms expressing the translational construct *Ptrx-3::trx-3::GFP* grown on *P. luminescens* display higher levels of the fluorescent reporter compared to the control animals grown on *E. coli* OP50 or on *S. marcescens* Db10. The fluorescence of transgenic animals grown on *E. coli* OP50 was set to a value of 1 and the values of transgenic animals grown on *S. marcescens* Db10 and *P. luminescens,* denoted in the upper right corner of each GFP image, indicate the fold induction±SD compared to the *E. coli* OP50 control. (C) Quantification of *trx-2, trx-3, lys-2, glrx-21,* and *glrx-22* mRNA levels by qPCR of N2 wild-type animals grown on *E. coli* OP50 and *P. luminescens* Hb and of *trx-3(tm2820)* animals grown on *P. luminescens* Hb. Bars represent the percentage of each mRNA species±SEM in the two different genetic backgrounds calculated from three independent assays. Two-tailed Student *t* test was used to determine statistical significance (^⁎^*p* < 0.05; ^⁎⁎⁎^*p* < 0.001), (D, E) The survival of the *trx-3(tm2820)* mutant after infection by (D) *S. marcescens* Db10 and (E) *P. luminescens* Hb compared to that by nonpathogenic *E. coli* OP50 was assayed at 25 °C. Kaplan–Meier plots were used to show the fraction of animals that survived against time. Infection survival assays were performed three times, obtaining similar results, and one representative experiment is shown. The survival rate of *trx-3(tm2820)* mutants was compared to that of the N2 wild-type control using the log-rank (Mantel–Cox) test and was not significant in both cases (*p* > 0.05). (F, G) The survival of the *trx-3-*overexpressing strain after infection by (F) *S. marcescens* Db10 and (G) *P. luminescens* Hb was performed as described above for the *trx-3(tm2820)* mutant. Two independent experiments were performed and one representative experiment is shown. The survival rate of the *trx-3*-overexpressing strains was compared to that of the N2 wild-type control using the log-rank (Mantel–Cox) test and was significantly different in both cases (*p* < 0.05).

The restricted expression of *trx-3* in *C. elegans* intestinal cells prompted us to examine the effects of different pathogens on *trx-3* mRNA expression (Supplementary Table 5). From this survey, we found that *trx-3* mRNA is induced upon infection by the bacteria *P. luminescens* Hb and the fungi *C. albicans* [25,42]. These results, together with our own killing assays with *P. aeruginosa* PA14 (Fig. 5B), suggest that *trx-3* has a role in response to specific pathogens. To further explore the role of *trx-3* in the infection by these pathogens we first confirmed by qPCR the *trx-3* mRNA induction upon exposure to *P. luminescens* but not *S. marcescens* (Fig. 6A) as previously reported [25]. This induction also occurs at the protein level (Fig. 6B) and seems to be specific for *trx-3*. Hence, the mRNA levels of other related members of the thioredoxin and glutaredoxin families expressed in the intestine, such as *trx-2, glrx-21,* and *glrx-22,* are not altered either in wild-type or *trx-3(tm2820)* mutant backgrounds (Fig. 6C). It is important to note that *trx-3* mRNA induction upon *P. luminescens* infection is similar to that of the lysozyme *lys-2,* a well-known marker for intestinal infection in *C. elegans* [43]. Indeed, *lys-2* induction is further enhanced in a *trx-3(tm2820)* background, suggesting that worms lacking *trx-3* are already sensitized to *P. luminescens* infection (Fig. 6C), whereas *trx-3(tm2820)* mutants grown on the nonpathogenic OP50 bacteria have 25% lower levels of *lys-2* compared to the wild-type control (data not shown). However, similar to what we have found with *P. aeruginosa* infection (Fig. 5B), *trx-3(tm2820)* mutants do not display enhanced sensitivity to killing by *P. luminescens* or *S. marcescens* (Figs. 6D and E), probably because of the induction of *lys-2* and other antimicrobial defenses. In turn, when overexpressed, TRX-3 significantly increased the survival of animals infected with *P. luminescens* but not with *S. marcescens* (Figs. 6F and G). (As no antibodies are available for *C. elegans* TRX-3, we were unable to evaluate the increase in total TRX-3 in *vzEx96-*overexpressing worms compared to wild-type controls. However, we determined the *trx-3* mRNA levels by qPCR, which resulted in a 70.92±6.73-fold induction (mean±SEM, two independent experiments with three replicates each)).

**Fig. 7:**
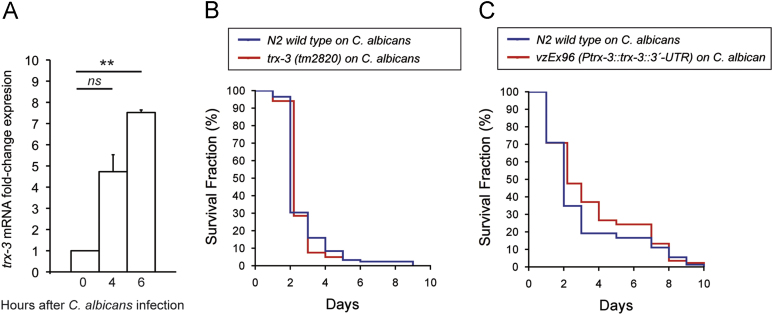
(A) Quantification of *trx-3* mRNA levels by qPCR of N2 wild-type animals grown on *E. coli* OP50 and *C. albicans*. Bars represent the average fold change in expression of two independent assays±SEM. Two-tailed Student *t* test was used to determine statistical significance (ns,= not significant; ^⁎⁎^*p* < 0.01). (B, C) The survival of (B) the *trx-3(tm2820)* mutant and (C) the *trx-3*-overexpressing strain after infection by *C. albicans* compared to that by nonpathogenic *E. coli* OP50 was assayed at 25 °C. Kaplan–Meier plots were used to show the fraction of animals that survive against time. Infection survival assays were performed two times, obtaining similar results, and one representative experiment is shown. The survival rates of the *trx-3(tm2820)* mutants and *trx-3*-overexpressing strain were compared to that of the N2 wild-type control using the log-rank (Mantel–Cox) test and was not significant in both cases (*p* > 0.05).

**Fig. 8:**
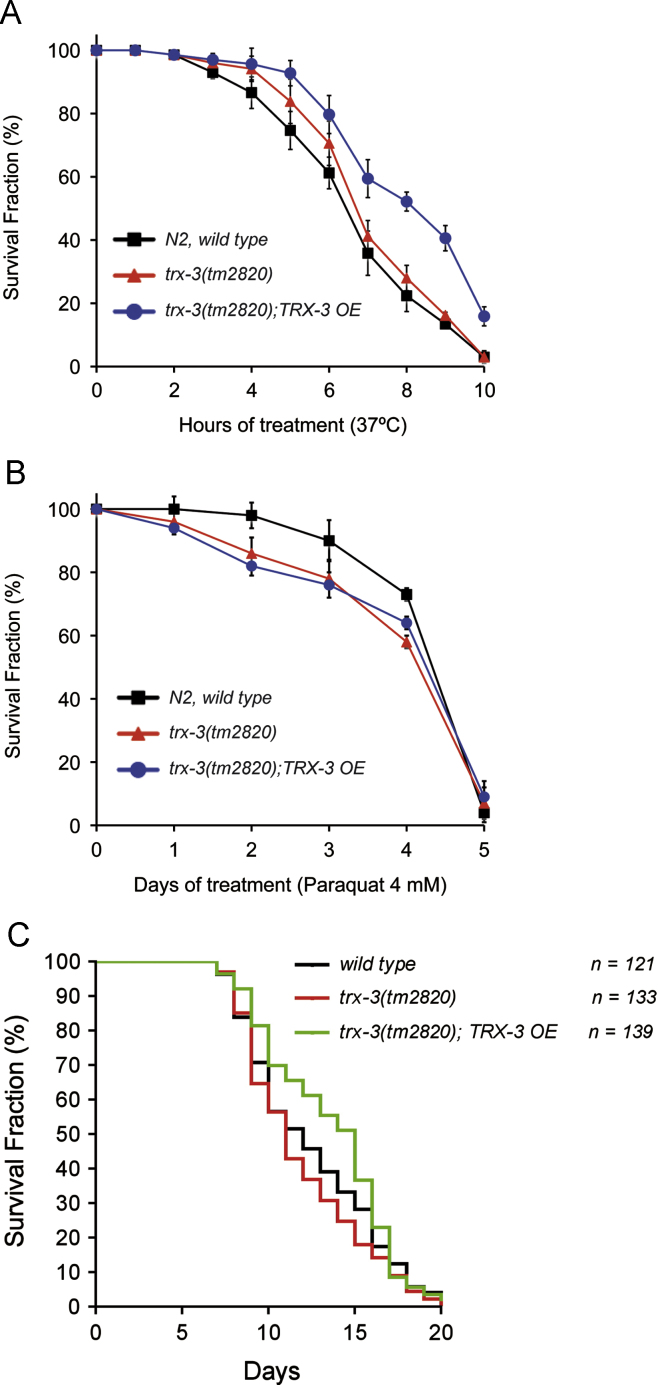
(A, B) N2 wild-type, *trx-3(tm2820)* mutant, and *trx-3*-overexpressing (OE) worms from the *vzEx96* array were assayed for their resistance to (A) heat shock at 37 °C and (B) paraquat 4 mM. Graphs represent the average of two independent experiments. The paraquat experiments were carried out at 20 °C. Error bars indicate the SEM. Differences between strains were not significant in all cases by one-way ANOVA (*p* > 0.05). (C) Longevity of *trx-3(tm2820)* mutants and *trx-3*-OE worms from the *vzEx96* array on *E. coli* OP50 was assayed at 25 °C. Kaplan–Meier plots were used to show the fraction of animals that survived over time. Longevity assays were performed twice, obtaining similar results, and the composite data are shown. The survival rate was obtained using the log-rank (Mantel–Cox) test and the differences were not significant in all cases (*p* > 0.05).

*trx-3* mRNA expression is also rapidly induced upon exposure to the fungus *C. albicans* [42] but not upon infection by other fungi such as *Drechmeria coniospora* and *Harposporium* sp. [25] (Supplementary Table 5). Although, in consonance with previous microarray analyses [42], we have confirmed that the induction of *trx-3* mRNA expression occurs rapidly (4–6 h) after exposure to *C. albicans* (Fig. 7A), *trx-3(tm2820)* mutants do not display enhanced sensitivity to *C. albicans* killing (Fig. 7B). However, similar to *P. luminescens,* there was a suggestion that overexpression of TRX-3 affords some slight protection against *C. albicans*-mediated killing. Although, in the case of *C. albicans,* this increased resistance was not statistically significant (Fig. 7C), as a whole, our data indicate that TRX-3 may protect against infection by certain specific pathogens. Finally, we asked whether overexpression of TRX-3 might also have a protective effect against stress or on worm longevity. As shown in Fig. 8, high levels of TRX-3 produced a small, although not significant, increase in life span and resistance against heat stress but had no effect on paraquat resistance. This is consistent with other data (Figs. 6G and 7C) suggesting that TRX-3 does not play a major stress-protective role in *C. elegans.*

## Discussion

Thioredoxins play a key role in the maintenance of redox homeostasis in most organisms and, therefore, this family of proteins is well conserved throughout evolution. As the organism complexity increases from bacteria to metazoa, three levels of diversity are identified within the thioredoxin family: (1) tissue/organ-specific expression, (2) specific subcellular localizations, and (3) proteins containing one or more thioredoxin modules within a multidomain protein organization. In mammals, tissue-specific thioredoxins have only been identified in spermatozoa and lung ciliated epithelial cells [44]. Germ-cell-specific expression of thioredoxins has also been reported in *Drosophila* [45]. Interestingly, we have identified novel tissue-specific expression patterns in the *C. elegans* thioredoxin family. Hence, TRX-1 is exclusively expressed in ASJ neurons [3] and TRX-2 is expressed in the mitochondria of ASEL and AIYL/R neurons and muscle cells under nonstress conditions and also in intestinal cells upon induction of the mitochondrial unfolded protein response [6]. We report here the characterization of *C. elegans* TRX-3, which, to our knowledge, is the first metazoan thioredoxin with a tissue-specific expression pattern restricted to intestine.

*trx-3(tm2820)* loss-of-function mutants do not show any gross developmental or morphological phenotypes or possess any phenotypes related to intestinal function under normal growth conditions. A possible explanation for the absence of a major phenotype in *trx-3(tm2820)* mutants could be a functional redundancy with other thioredoxins or closely related proteins such as glutaredoxins [31]. We have ruled out this possibility for *trx-1* and *trx-2,* as double mutants of these two genes with *trx-3(tm2820)* are also viable, with no apparent phenotype. Moreover, we failed to identify any synthetic interaction when downregulating all known members of the thioredoxin, glutaredoxin, and peroxiredoxin protein families in a *trx-3(tm2820)* mutant background. As RNAi feeding penetrance can be highly variable depending on genetic backgrounds and tissue expression, combinations of other thioredoxin, glutaredoxin, and peroxiredoxin system mutants with that of *trx-3* will be needed to unequivocally identify the redundant system. In addition, we cannot rule out that the redundant system could be a more distant member of the thioredoxin/glutaredoxin family not included in our RNAi screen. Alternatively, the absence of phenotype of *trx-3(tm2820)* mutants could be also explained by the possibility that the aberrant ΔTRX-3 protein produced by the *trx-3(tm2820)* allele could retain some of the wild-type TRX-3 functionality despite being inactive in enzymatic activity assays, in vitro. Finally, the lack of phenotype might reflect a nonessential function of *trx-3* under nonstressed conditions.

Therefore, we asked if *trx-3* might be required when the animals are stressed. This idea made sense, as the worm intestine (together with the hypodermis) is directly exposed to variable and sometimes harsh environmental conditions [11] and because thioredoxins have been long known to have a protective function against various types of stress [31]. However, *trx-3(tm2820)* mutants did not show enhanced sensitivity to heat shock or oxidative stress treatments and did not induce expression of intestinal stress markers. Intestinal cells are also the main target of bacterial and fungal pathogens and, indeed, *trx-3* seems to be specifically upregulated upon infection by certain bacterial pathogens. such as *P. luminescens* Hb [25], but not by other bacteria such as *P. aeruginosa* PA14 [46], *S. marcescens* Db10, *Enterococcus faecalis* [25], *Staphylococcus aureus* RN6390 [47], or *Bacillus thuringiensis* NRRL B-18247 [43]. Similarly, *trx-3* mRNA expression is induced upon *C. albicans* infection [42] but not upon other fungal infections such as *D. coniospora* and *Harposporium* sp. [25] (Supplementary Table 5).

The induction of *trx-3* expression after *P. luminescens* and *C. albicans* infection is very rapid ([25,42] and Figs. 6A and 7A) and, interestingly, in the case of *C. albicans,* this *trx-3* upregulation is independent of the infective capacity of the pathogen, as heat-killed *C. albicans* (which is avirulent) induces *trx-3* mRNA expression to an extent similar to live *C. albicans* [42]. These data point to the possibility that *trx-3* is induced in response to the presence of specific molecules on the surface of the pathogen (named pathogen-associated molecular patterns, PAMPs). PAMP recognition is the earliest event in the *C. elegans* response against pathogen infection, which is triggered independent of its infective capabilities [48]. Consistently, TRX-3 overexpression provides some protection for worms against *P. luminescens* and possibly *C. albicans* infection. This suggests that the induction of TRX-3 could have a protective function during early stages of the infection. Although these data are consistent with a protective role for TRX-3 in the early stages of infection, the similar sensitivities of *trx-3(tm2820)* mutants and wild-type animals to killing by these pathogens suggests that other TRX-3-independent mechanisms may be more important in preventing killing during the later stages of infection. Yet, given the number of downstream effectors induced upon infection and the multifactorial nature of the immune response, further work is needed to determine the real impact of *trx-3* in the *C. elegans* response to pathogen infection, as the lack of one single effector can have no impact on nematode survival owing to the induction of other antimicrobial defenses. Indeed, we have found that *lys-2* is induced in *trx-3(tm2820)* mutants (Fig. 6C), which might compensate for the loss of *trx-3,* along with other unidentified protective genes.

TRX-3 protein is composed of 158 amino acid residues, contains the conserved redox active-site WCGPC, and, surprisingly, appears to be restricted to the *Caenorhabditis* genus. Interestingly, a phylogenetic analysis demonstrated that TRX-3 clusters with invertebrate and vertebrate nucleoredoxins and nucleoredoxin-like proteins [49]. Nucleoredoxins are typically of a size similar to that of *C. elegans* TRX-3 but differ in the sequence of their redox active site in that the intervening residues between the two cysteines are different from those of classical thioredoxins. In turn, the previously described *C. elegans* TRX-1 and TRX-2 [3,6] are found clustering together with yeast, *Drosophila,* and human thioredoxins in a different branch of the phylogenetic tree [49]. Thus, given the close phylogenetic proximity of *C. elegans* TRX-3 with nucleoredoxins and related proteins, a possible role of vertebrate nucleoredoxins in host defense against infection arises as an interesting hypothesis. This idea further strengthens the relevance of our findings in *C. elegans* by its potential application to human pathologies.

Nematodes produce reactive oxygen species (ROS) by the dual oxidase Ce-Duox1/BLI-3 as a defensive mechanism against diverse pathogen infections [50,51]. Unavoidably, this ROS production also causes a cellular damage in the host tissue, which is counteracted by the induction of a general oxidative stress response mainly orchestrated by the SKN-1 transcription factor [52,53]. Despite thioredoxins being considered general antioxidant enzymes [31], it is unlikely that *trx-3* is part of this protective mechanism as *trx-3(tm2820)* mutants are as sensitive as wild-type animals to killing by *P. luminescens* and *C. albicans*, and *trx-3(tm2820)* mutants do not show enhanced sensitivity to ROS-producing chemicals such as juglone or paraquat. Moreover, as mentioned above, avirulent, heat-killed *C. albicans* also induces *trx-3* to an extent similar to that achieved by the infective, live fungus. However, we cannot rule out other roles for *trx-3* in gut immunity, such as the establishment of the necessary redox environment for an efficient immune response or a direct effect toward microbial virulence factors.

Several signaling pathways have been identified to mediate the intestinal response to pathogen infection. These pathways include the insulin-like, TGF-β, Toll receptor, and P38, ERK, and JNK MAPK pathways as well as pathways regulated by ELT-2 and HSF-1 transcription factors [54]. It is now well established that *C. elegans* elicits distinct responses depending on the pathogen to which it is exposed [55], although the mechanisms underlying this differential response are still poorly understood. For instance, *C. elegans* infection by *C. albicans* has been shown to mainly induce genes under the control of the p38 MAPK signaling pathway [42], whereas *P. luminescens* infection causes a significant elevation of genes under the control of both P38 MAPK and TGF-β pathways [55]. We have found that *trx-3* expression is regulated by *dbl-1,* a TGF-β ligand involved in worm size control, development of male tail structures, and worm innate immunity [26,27]. Interestingly, *trx-3* appears to be tightly regulated by DBL-1 levels as both *dbl-1* mutation and *dbl-1* overexpression cause a significant *trx-3* downregulation. Thus, it is plausible that *trx-3* induction in response to *P. luminescens* or *C. albicans* exposure may be controlled by the TGF-β signaling pathway.

In summary, we describe here the functional characterization of the first metazoan thioredoxin with an expression pattern restricted to intestinal cells. Although no major phenotype has been found in *trx-3* RNAi-downregulated worms or *trx-3(tm2820)* mutants, the fact that *trx-3* is induced upon exposure to specific bacterial and fungal pathogens suggests that TRX-3 could be part of the molecular mechanism mounted to counteract this insult. Our results identify TRX-3 as a promising candidate for delving further into the molecular pathways governing the differential innate immune response of *C. elegans* and, by extrapolation, the role of mammalian thioredoxins in intestinal function and pathogen infection.

## Figures and Tables

**Table 1:** Developmental parameters of *trx-3(tm2820)* mutants.

Developmental parameter	Wild type	*trx-3(tm2820)*	*p* (unpaired two-tailed *t* test)
Progeny size^a^	269.8±35.4	262.9±24.4	0.6188
Defecation cycle timing (pBoc)^b^	7.796±1.11	6.005±1.12	0.0019
Length (arbitrary units)^c^	590.5±8.5	496.0±56.5	<0.0001
Time of egg-laying (h after L1 refeeding)^d^	56.3±1.23	55.9±2.09	0.7866

a The mean brood size±standard deviation (SD) from 20 worms of each genotype was determined.

b The pBoc (peak of the posterior body muscle contraction) was monitored at 20 °C in L4 animals crawling on a lawn of OP50 bacteria on agar plates. pBoc rhythmicity of individual worms was determined by calculating the coefficient of variance (CV), which is defined as the standard deviation of the pBoc period expressed as percentage of the mean from 12 successive contractions. The data represent the CV mean±SD of six independent trials, each composed of 20 independent animals.

c The length of the worms was determined using the COPAS Biosorter. The mean length±SD of three independent trials with approximately 500 worms of each genotype is shown. This phenotype was not caused by a delayed growth, as demonstrated by a similar egg-laying time between wild-type and *tm2820* animals.

d L1 synchronized larvae (generated by a 16-h incubation in M9 buffer of hypochlorite-purified eggs) were grown at 20 °C on OP50 agar plates and the time at which the first eggs were laid was determined. The mean of the egg-laying time±SD from 30 worms of each genotype is shown.
