# Supplementary material for: Functional characterization of thioredoxin 3 (TRX-3), a Caenorhabditis elegans intestine-specific thioredoxin
Source: Free Radic Biol Med. 2014 Mar;68(100):205–19. doi: 10.1016/j.freeradbiomed.2013.11.023 (PMC4018987; doi:10.1016/j.freeradbiomed.2013.11.023)
Supplement: Supplementary file 1 — Supplementary Material [file mmc1.doc]

Supplementary Table 1: Strains used in this study

| *Strain name* | *Genotype* | *Reference/Source* |
| --- | --- | --- |
|  |  |  |
|  | *Basic strains* |  |
| N2 | *Wild type, DR subclone of CB original (Tc1 pattern I)* | CGC^a^ |
| VZ1 | *trx-1(ok1449) Ii* | [1] |
| VZ13 | *trx-2(tm2720) V* | [2] |
| VZ68 | *trx-3(tm2820) IV* | This study |
| VZ114 | *trx-1(ok1449) II; trx-3(tm2820) IV* | This study |
| VZ335 | *trx-3(tm2820) IV; trx-2(tm2720) V* | This study |
| NU3 | *dbl-1(nk3) V* | CGC |
| TK22 | *mev-1(kn1) III* | [3] |
| HB508 | *cav-2(tm394) V* | [4] |
| VZ145 | *trx-3(tm2820) IV; cav-2(tm394) V* | This study |
| VE1 | *prdx-2(gk169) II* | [25] |
|  | *trx-3 reporter and overexpressing strains* |  |
| VZ119 | *vzEx32 [pVZ35 (Ptrx-3::gfp); pRF4 (rol-6(su1006))]* | This study |
| VZ132 | *vzEx36 [pVZ47 (Ptrx-3::trx-3::gfp); pRF4 (rol-6(su1006)]* | This study |
| VZ262 | *trx-3(tm2820) IV; vzEx96 [Ptrx-3::trx-3::trx-3 3´-UTR; Punc-122::gfp]* | This study |
| VZ177 | *vzEx56 [pVZ374 (Ptrx-3::mCherry); Punc-122::gfp]* | This study |
| ENL10 | *dbl-1(nk3) V; vzEx56 [pVZ374 (Ptrx-3::mCherry); Punc-122::gfp]* | This study |
| ENL11 | *ctIs40 [ZC421(dbl-1(+)); pTG98 (Psur-5::gfp)] X;* *vzEx56 [pVZ374 (Ptrx-3::mCherry); Punc-122::gfp]* | This study |
|  |  |  |
|  | *Intestinal apical membrane reporter strains* |  |
| KWN117 | *pha-1(e2123) III; him-5(e1490) V; rnyEx60 [pELA2 (Pvha-6::vha-6::mCherry); Pmyo-3::gfp; pCL1 (pha-1+)]* | CGC |
| VZ213 | *pha-1(e2123) III; trx-3(tm2820) IV; him-5(e1490) V; rnyEx 60 [pELA2 (Pvha-6::vha-6::mCherry); Pmyo-3::gfp; pCL1 (pha-1+)]* | This study |
| HB157 | *jwEx157 [pSP014 (Pcav-2::cav-2::yfp)]* | [4] |
| VZ236 | *trx-3(tm2820) IV; jwEx157 [pSP014 (Pcav-2::cav-2::yfp)]* | This study |
|  |  |  |
|  | *Stress reporter strains* |  |
| XG88 | *gpEx88 [Pdaf-16::daf-16 a::gfp; pRF4 (rol-6(su1006))]* | [5] |
| VZ156 | *trx-3(tm2820) IV; gpEx88 [Pdaf-16::daf-16 a::gfp; pRF4 (rol-6(su1006))]* | This study |
| CL2070 | *dvIs70 [pCL25 (Phsp16.2::gfp); pRF4 (rol-6(su1006))]* | [6] |
| VZ210 | *trx-3(tm2820) IV; dvIs70 [pCL25 (Phsp16.2::gfp); pRF4 (rol-6(su1006))]* | This study |
| CL2166 | *dvIs19 [pAF15 (Pgst-4::gfp::NLS); pRF4 (rol-6(su1006))]* | [7] |
| VZ197 | *trx-3(tm2820) IV; dvIs19 [pAF15 (Pgst-4::gfp::NLS); pRF4 (rol-6(su1006))]* | This study |
|  |  |  |
|  | *daf-2 strains* |  |
| CB1370 | *daf-2(e1370) III* | CGC |
| VZ156 | *daf-2(e1370) III; trx-3(tm2820) IV* | This study |
| DH26 | *fer-15(b26) II* | CGC |
| GM6 | *fer-15(b26) II; daf-2(e1370) III* | gift, Manuel Muñoz |
| VZ211 | *fer-15(b26) II; trx-3(tm2820) IV* | This study |
| VZ212 | *fer-15(b26) II; daf-2(e1370) III; trx-3(tm2820) IV* | This study |
|  |  |  |
|  | *rrf-3 strains* |  |
| NL2099 | *rrf-3(pk1426) II* | [8] |
| VZ88 | *rrf-3(pk1426) II; trx-3 (tm2820) IV* | This study |
|  | *HyPer strains* |  |
| JRIS1 | *jrIs1(Prpl-17::HyPer* | [9] |
| VZ479 | *trx-3 (tm2820) IV; jrIs1(Prpl-17::HyPer* | This study |

^a^ Caenorhabditis Genetics Center (<http://www.cbs.umn.edu/CGC/>)

Supplementary Table 2: Primers used in RT-PCR and qPCR analyses

| *Gene name* | *Primer sequence* | *Orientation* |
| --- | --- | --- |
|  |  |  |
| *trx-3* | 5´-ATGGCTAAGAACTTTTTCTCCG- 3´ | forward |
|  | 5´-TTATGCACGGATTCTCTCGAG- 3´ | reverse |
|  |  |  |
| *ama-1* | 5´- ttccaagcgccgctgcgcattgtc -3´ | forward |
|  | 5´- cagaatttccagcactcgaggagcgga -3´ | reverse |
|  |  |  |
| *act-1* | 5´- CCATCATGAAGTGCGACATTG -3´ | forward |
|  | 5´- CATGGTTGATGGGGCAAGAG -3´ | reverse |
|  |  |  |
| *lys-2* | 5´- CAGTCTGGATTCAGGTTACTTCCC -3´ | forward |
|  | 5´- ACCACCTCCAAGAACATTCC -3´ | reverse |
|  |  |  |
| *trx-2* | 5´- GACTCGAGGAAAAGGTAAACGGA-3´ | forward |
|  | 5´- ATACCGTTGGCACCGCAGAG-3´ | reverse |
|  |  |  |
| *glrx-21* | 5´- AAGGATCCAGTTGTGATGTACACA-3´ | forward |
|  | 5´- GACAAAGATCTGTGGCACACTGG-3´ | reverse |
|  |  |  |
| *glrx-22* | 5´- GTTGAGGAAGTCAAGGAACATGCG-3´ | forward |
|  | 5´- GGAACAGTTCGCTGACGTGTC-3´ | reverse |
|  |  |  |

Supplementary Table 3: Description of the *C. elegans* genes coding for thioredoxins with the conserved active site sequence WCGPC.

| *Gene name* | *Gene sequence designation* | *Amino acid*  *residues* | *Domain organisation* | *Reference* |
| --- | --- | --- | --- | --- |
|  |  |  |  |  |
| *trx-1* | *B0228.5* | 115 / 114^a^ | 1 thioredoxin domain | [1] |
| *trx-2* | *B0024.9* | 145 | 1 thioredoxin domain | [2] |
| *trx-3* | *M01H9.1* | 158 | 1 thioredoxin domain | This study |
| *trx-4* | *Y44E3A.3* | 107 | 1 thioredoxin domain | Not reported |
| *trx-5* | *K02H11.6* | 142 | 1 thioredoxin domain | Not reported |
| *txl* | *Y54E10A.3* | 284 | 1 N-terminal thioredoxin domain  followed by 1 proteasome interacting domain | Not reported |
| *dnj-27* | *Y47H9C.5* | 788 / 318^b^ | 1 N-terminal DNAJ domain followed by four thioredoxin domains whereby the last one is the only having a WCGPC active sequence | [10] |
| *png-1* | *F56G4.5* | 606 | 1 N-terminal thioredoxin domain  followed by a domain of unknown function | [11] |
| *-* | *Y55F3AR.2* | 254 | 1 N-terminal thioredoxin domain  followed by a domain of unknown function | Not reported |

^a^Both splice variants verified experimentally [1]

^b^Shorter splice variant not verified experimentally (F. Muñoz-Lobato, unpublished observation).

Supplementary Table 4: Genes tested in RNAi feeding assays for visible synthetic defects with *trx-3(tm2820)* mutants^a^.

| *Protein Family* | *Gene name* | *Gene sequence*  *designation* | *Synthetic*  *phenotype* |
| --- | --- | --- | --- |
|  |  |  |  |
| *thioredoxins* | *trx-1* | B0228.5 | No |
|  | *trx-2* | B0024.9 | No |
|  | *trx-3* | M01H9.1 | No |
|  | *txl* | Y54E10A.3 | No |
|  | *dnj-27* | Y47H9C.5 | No |
|  | *trx-4* | Y44E3A.3 | No |
|  | *trx-5* | K02H11.6 | No |
|  |  |  |  |
| *thioredoxin reductases* | *trxr-1* | C06G3.7 | No |
|  | *trxr-2* | ZK637.10 | No |
|  |  |  |  |
| *glutaredoxins* | *glrx-5* | Y49E10.2 | NA^b^ |
|  | *glrx-10* | Y34D9A.6 | No |
|  | *glrx-21* | ZK121.1 | No |
|  | *glrx-22* | C07G1.8 | No |
|  |  |  |  |
| *peroxiredoxins* | *prdx-2* | F09E5.15 | NA^b^ |
|  | *prdx-3* | R07E5.2 | No |
|  | *prdx-6* | Y38C1AA.11 | No |
|  |  |  |  |
| *glutathione reductase* | *gsr-1* | C46F11.2 | No |

^a^ All genes were tested by conducting RNAi feeding with the indicated gene in both *rrf-3(pk1426)* and *rrf-3(pk1426); trx-3(tm2820)* genetic backgrounds. The *rrf-3*(*pk1426)* mutation increases the sensitivity to RNAi [8].

^b^ Not applicable. RNAi of these genes caused larval arrest/slow growth in the *rrf-3(pk1426)* control strain.

Supplementary Table 5: Genome-wide studies where *trx-3* mRNA levels have been determined upon pathogen infection.

| *Pathogen* | *Differentially expressed versus OP50* | *Fold induction (+/-)* | *p value* | *Reference* |
| --- | --- | --- | --- | --- |
|  |  |  |  |  |
| *Gram Positive Bacteria* |  |  |  |  |
|  |  |  |  |  |
| *Bacillus* thuringiensis ***NRRL B-18247*** | No | <2 | >0.05 | [12] |
| *Enterococcus faecalis OG1RF* | **YES** | 1.9^b^ | Not specified | [13] |
| *Microbacterium nematophilum CBX102* | No | <2 | >0.05 | [14] |
| *Staphylococcus aureus RN6390* | No | <2 | >0.01 | [15] |
|  |  |  |  |  |
| *Gram Negative Bacteria* |  |  |  |  |
|  |  |  |  |  |
| *Photorhabdus luminescens Hb* | **YES** | 4.55^b^ | Not specified | [13] |
| *Pseudomonas aeruginosa PA14* | No | <2 | >0.01 | [16] |
| *Pseudomonas aeruginosa PA14 in octr-1 mutant background* | **YES** | 4.25 | 0.0084 | [17] |
| *Serratia marcescens Db10* | No | 0.258 | Not specified | [13] |
| *Vibrio cholerae O1* | No | <1.2 | >0.05 | [18] |
| *Yersinia pestis KIM5* | No | <2 | >0.05 | [19] |
|  |  |  |  |  |
| *Fungi* |  |  |  |  |
|  |  |  |  |  |
| *Candida albicans (alive)* | **YES^a^** | 9.6 | <E-10 | [20] |
| *Candida albicans (heat-killed)* | **YES^a^** | 13.3 | <E-16 | [20] |
| Drechmeria coniospora | No | 0.50 | Not specified | [13] |
| Harposporium sp. | No | 0.90 | Not specified | [13] |
|  |  |  |  |  |

*^a^*On heat-killed OP50

^b^log2 ratio versus OP50

**Supplementary Figure Legends**

**Supplementary Figure 1**. (A) Alignment of the amino acid sequence of *C. elegans* TRX-3 with that of *C. elegans* TRX-1a [1]*, C elegans* TRX-2 [2], human TRX-1 [21] and human TRX-2 [22]. Identical residues are shown in black boxes while similar residues are shown in grey. The active site sequence WCGPC is shown in a red box. The Megalign Software integrated in the Lasergene Suite Package (DNASTAR) was used for alignment of the sequences by the Clustal W method [23]. (B) Comparison of the predicted truncated ΔTRX-3 protein (red) encoded by the *trx-3(tm2820)* allele with that encoded by the *trx-3* wild-type allele. The position of the introns is shown by inverted triangles and the active site is boxed. Numbers on the right indicate the amino acid residues. Note that the truncated ΔTRX-3 protein resulting from expression of the *tm2820* allele is half the size of the wild-type mature TRX-3 protein. (C) Theoretical 3D-model of *C. elegans* wild-type TRX-3 and ΔTRX-3 from the *tm2820* allele. 3D-models were generated using the Swiss-Model Proteomic Server [24]. Orthologous human TRX-1 was also modelled as a control. Note the strong similarity of 3D-structure between human TRX-1 and *C. elegans* TRX-3 proteins, which is severely modified in that of the ΔTRX-3 from the *tm2820* allele.

**Supplementary Figure 2.** (A-B) Longevity of *trx-1(ok1449); trx-3(tm2820)* (A) and *trx-3(tm2820); trx-2(tm2720)* (B) double mutants on *E. coli* OP50 was assayed at 25ºC. Kaplan-Meier plots were used to show the fraction of animals that survive over time. Longevity assays were performed twice, obtaining similar results, and the composite data is shown. The survival rate of *trx-3(tm2820)* animals was compared to that of their respective controls using the log-rank (Mantel-Cox) test and the differences were found not significant in all cases (*p*>0.05). When comparing *trx-1* backgrounds versus their corresponding non *trx-1* controls, the differences were highly significant (*** *p*<0.001).

**Supplementary Figure 3. (**A) The redox state of PRDX-2 was examined in *wild-type* (N2) and *trx-3(tm2820)* worms before and following treatment with 1mM H_2_O_2_ for 5 minutes. Equal amounts of proteins extracted from approximately 3000 *wild-type* (N2), *trx-3(tm2820)* or *prdx-2(gk169)* animals (and normalized using the bicinchoninic acid assay) were separated by non-reducing SDS-PAGE followed by Western blot analysis with anti-PRDX-2 antibodies [25]. This analysis allows electrophoretic separation and identification of reduced (monomeric) and oxidized (dimeric) PRDX-2, whose levels are not different in *trx-3(tm2820)* mutants compared with wild type controls. The absence of these bands in extracts from *prdx-2(gk169)* worms demonstrates the specificity of the anti-PRDX-2 antibodies. Treatment with 5% β-mercaptoethanol (β-ME) for 5 minutes results in complete reduction of PRDX-2 disulfide dimers. (B-C) Determination of the H_2_O_2_ levels in *trx-3(tm2820)* worms compared to wild type controls measured as the ratio oxidized/reduced HyPer [9]. (B) A representative example of the fluorescence of *jrIs1(Prpl-17::HyPer)* and *trx-3(tm2820); jrIs1(Prpl-17::HyPer)* animals. The head of the worm is on the left. Bar 100 μm. (C) For quantification, 12 to 15 *jrIs1(Prpl-17::HyPer)* and *trx-3(tm2820); jrIs1(Prpl-17::HyPer)* young adults were imaged with the same settings and their fluorescence was measured throughout the whole worm body using the ImageJ software. Bars represent the mean fluorescence ± SD. Differences were non significant by a two-tail Student´s *t-*test (*p* >0.05).

**References**

[1] Miranda-Vizuete, A.; Fierro Gonzalez, J. C.; Gahmon, G.; Burghoorn, J.; Navas, P.; Swoboda, P. Lifespan decrease in a Caenorhabditis elegans mutant lacking TRX-1, a thioredoxin expressed in ASJ sensory neurons. *FEBS Lett* **580:**484-490; 2006.

[2] Cacho-Valadez, B.; Munoz-Lobato, F.; Pedrajas, J. R.; Cabello, J.; Fierro-Gonzalez, J. C.; Navas, P.; Swoboda, P.; Link, C. D.; Miranda-Vizuete, A. The characterization of the Caenorhabditis elegans mitochondrial thioredoxin system uncovers an unexpected protective role of TRXR-2 in beta-amyloid peptide toxicity. *Antioxid Redox Signal* **16:**1384-1400; 2012.

[3] Ishii, N.; Takahashi, K.; Tomita, S.; Keino, T.; Honda, S.; Yoshino, K.; Suzuki, K. A methyl viologen-sensitive mutant of the nematode Caenorhabditis elegans. *Mutat Res* **237:**165-171; 1990.

[4] Parker, S.; Walker, D. S.; Ly, S.; Baylis, H. A. Caveolin-2 is required for apical lipid trafficking and suppresses basolateral recycling defects in the intestine of Caenorhabditis elegans. *Mol Biol Cell* **20:**1763-1771; 2009.

[5] Henderson, S. T.; Johnson, T. E. daf-16 integrates developmental and environmental inputs to mediate aging in the nematode Caenorhabditis elegans. *Curr Biol* **11:**1975-1980; 2001.

[6] Link, C. D.; Cypser, J. R.; Johnson, C. J.; Johnson, T. E. Direct observation of stress response in Caenorhabditis elegans using a reporter transgene. *Cell Stress Chaperones* **4:**235-242; 1999.

[7] Link, C. D.; Johnson, C. J. Reporter transgenes for study of oxidant stress in Caenorhabditis elegans. *Methods Enzymol* **353:**497-505; 2002.

[8] Sijen, T.; Fleenor, J.; Simmer, F.; Thijssen, K. L.; Parrish, S.; Timmons, L.; Plasterk, R. H.; Fire, A. On the role of RNA amplification in dsRNA-triggered gene silencing. *Cell* **107:**465-476; 2001.

[9] Back, P.; De Vos, W. H.; Depuydt, G. G.; Matthijssens, F.; Vanfleteren, J. R.; Braeckman, B. P. Exploring real-time in vivo redox biology of developing and aging Caenorhabditis elegans. *Free Radic Biol Med* **52:**850-859; 2012.

[10] Munoz-Lobato, F.; Rodriguez-Palero, M. J.; Naranjo-Galindo, F. J.; Shepard, F.; Gaffney, C. J.; Szewczyk, N. J.; Hamamichi, S.; Caldwell, K. A.; Caldwell, G. A.; Link, C. D.; Miranda-Vizuete, A. Protective role of DNJ-27/ERdj5 in Caenorhabditis elegans models of human neurodegenerative diseases. *Antioxid Redox Signal* **in press. doi:10.1089/ars.2012.5051.**; 2013.

[11] Suzuki, T.; Tanabe, K.; Hara, I.; Taniguchi, N.; Colavita, A. Dual enzymatic properties of the cytoplasmic peptide: N-glycanase in C. elegans. *Biochem Biophys Res Commun* **358:**837-841; 2007.

[12] Boehnisch, C.; Wong, D.; Habig, M.; Isermann, K.; Michiels, N. K.; Roeder, T.; May, R. C.; Schulenburg, H. Protist-type lysozymes of the nematode Caenorhabditis elegans contribute to resistance against pathogenic Bacillus thuringiensis. *PLoS One* **6:**e24619; 2011.

[13] Engelmann, I.; Griffon, A.; Tichit, L.; Montanana-Sanchis, F.; Wang, G.; Reinke, V.; Waterston, R. H.; Hillier, L. W.; Ewbank, J. J. A comprehensive analysis of gene expression changes provoked by bacterial and fungal infection in C. elegans. *PLoS One* **6:**e19055; 2011.

[14] O'Rourke, D.; Baban, D.; Demidova, M.; Mott, R.; Hodgkin, J. Genomic clusters, putative pathogen recognition molecules, and antimicrobial genes are induced by infection of C. elegans with M. nematophilum. *Genome Res* **16:**1005-1016; 2006.

[15] Irazoqui, J. E.; Troemel, E. R.; Feinbaum, R. L.; Luhachack, L. G.; Cezairliyan, B. O.; Ausubel, F. M. Distinct pathogenesis and host responses during infection of C. elegans by P. aeruginosa and S. aureus. *PLoS Pathog* **6:**e1000982; 2010.

[16] Troemel, E. R.; Chu, S. W.; Reinke, V.; Lee, S. S.; Ausubel, F. M.; Kim, D. H. p38 MAPK regulates expression of immune response genes and contributes to longevity in C. elegans. *PLoS Genet* **2:**e183; 2006.

[17] Sun, J.; Singh, V.; Kajino-Sakamoto, R.; Aballay, A. Neuronal GPCR controls innate immunity by regulating noncanonical unfolded protein response genes. *Science* **332:**729-732; 2011.

[18] Sahu, S. N.; Lewis, J.; Patel, I.; Bozdag, S.; Lee, J. H.; LeClerc, J. E.; Cinar, H. N. Genomic analysis of immune response against Vibrio cholerae hemolysin in Caenorhabditis elegans. *PLoS One* **7:**e38200; 2012.

[19] Bolz, D. D.; Tenor, J. L.; Aballay, A. A conserved PMK-1/p38 MAPK is required in caenorhabditis elegans tissue-specific immune response to Yersinia pestis infection. *J Biol Chem* **285:**10832-10840; 2010.

[20] Pukkila-Worley, R.; Ausubel, F. M.; Mylonakis, E. Candida albicans infection of Caenorhabditis elegans induces antifungal immune defenses. *PLoS Pathog* **7:**e1002074; 2011.

[21] Wollman, E. E.; d'Auriol, L.; Rimsky, L.; Shaw, A.; Jacquot, J. P.; Wingfield, P.; Graber, P.; Dessarps, F.; Robin, P.; Galibert, F.; et al. Cloning and expression of a cDNA for human thioredoxin. *J Biol Chem* **263:**15506-15512; 1988.

[22] Damdimopoulos, A. E.; Miranda-Vizuete, A.; Pelto-Huikko, M.; Gustafsson, J. A.; Spyrou, G. Human mitochondrial thioredoxin. Involvement in mitochondrial membrane potential and cell death. *J Biol Chem* **277:**33249-33257; 2002.

[23] Thompson, J. D.; Higgins, D. G.; Gibson, T. J. CLUSTAL W: improving the sensitivity of progressive multiple sequence alignment through sequence weighting, position-specific gap penalties and weight matrix choice. *Nucleic Acids Res* **22:**4673-4680; 1994.

[24] Arnold, K.; Bordoli, L.; Kopp, J.; Schwede, T. The SWISS-MODEL workspace: a web-based environment for protein structure homology modelling. *Bioinformatics* **22:**195-201; 2006.

[25] Olahova, M.; Taylor, S. R.; Khazaipoul, S.; Wang, J.; Morgan, B. A.; Matsumoto, K.; Blackwell, T. K.; Veal, E. A. A redox-sensitive peroxiredoxin that is important for longevity has tissue- and stress-specific roles in stress resistance. *Proc Natl Acad Sci U S A* **105:**19839-19844; 2008.
