# Supplementary figures and images for: Functional characterization of thioredoxin 3 (TRX-3), a Caenorhabditis elegans intestine-specific thioredoxin
Source: Free Radic Biol Med. 2014 Mar;68(100):205–19. doi: 10.1016/j.freeradbiomed.2013.11.023 (PMC4018987; doi:10.1016/j.freeradbiomed.2013.11.023)

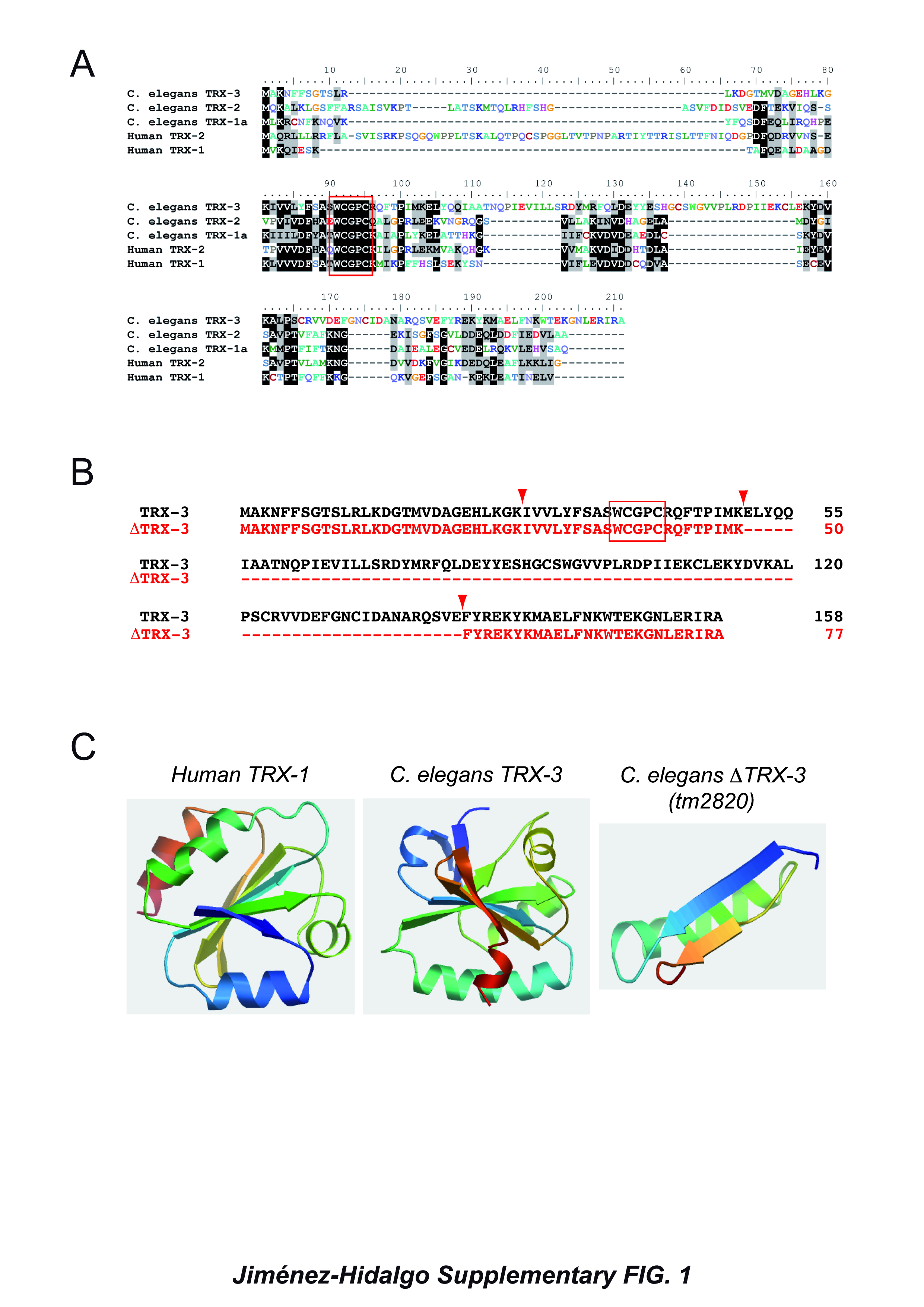

Supplement: Supplementary file 2 — Supplementary Material [file mmc2.zip › Supplementary Figure 1.tif]

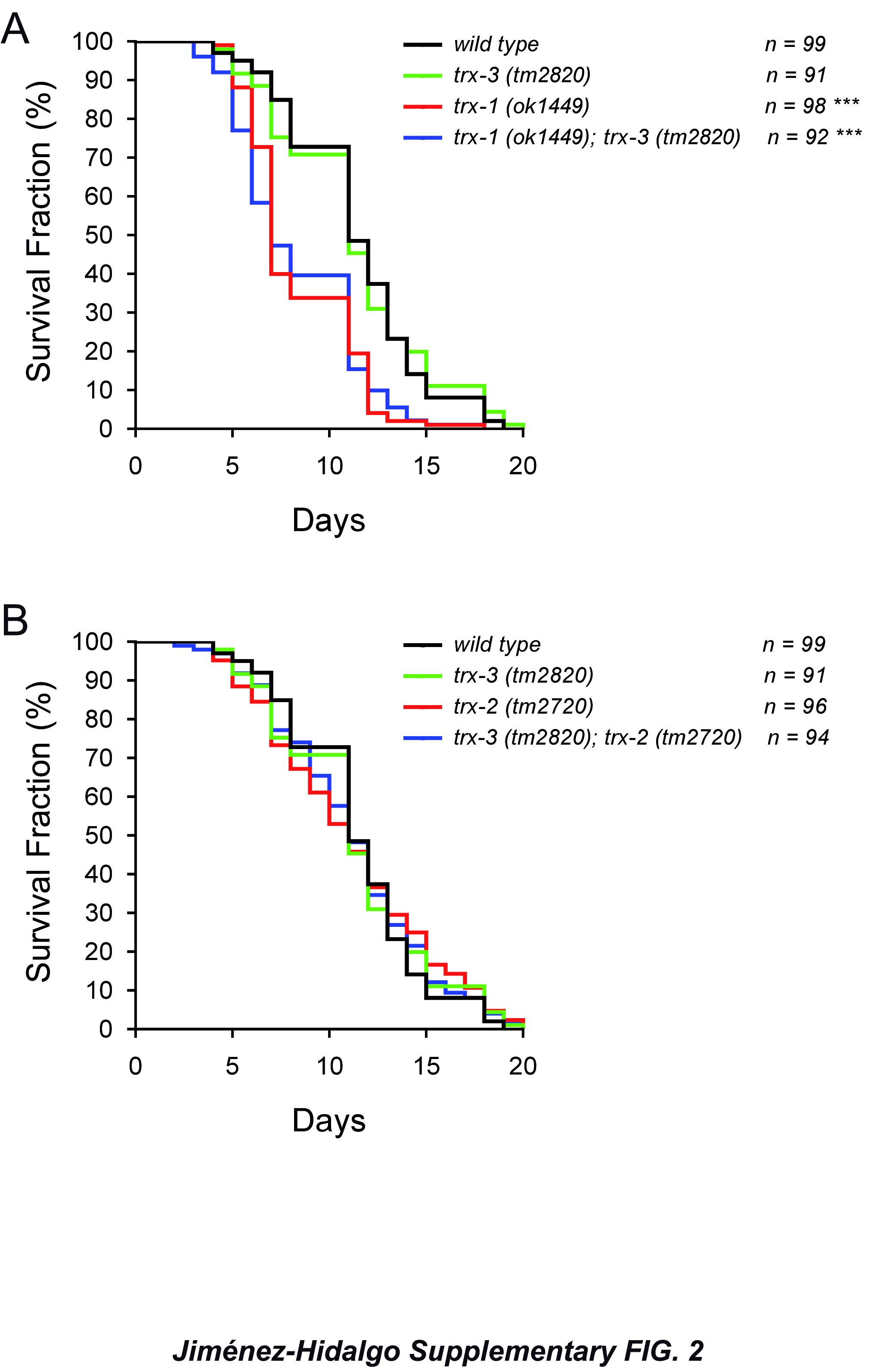

Supplement: Supplementary file 3 — Supplementary Material [file mmc3.zip › Supplementary Figure 2.tif]

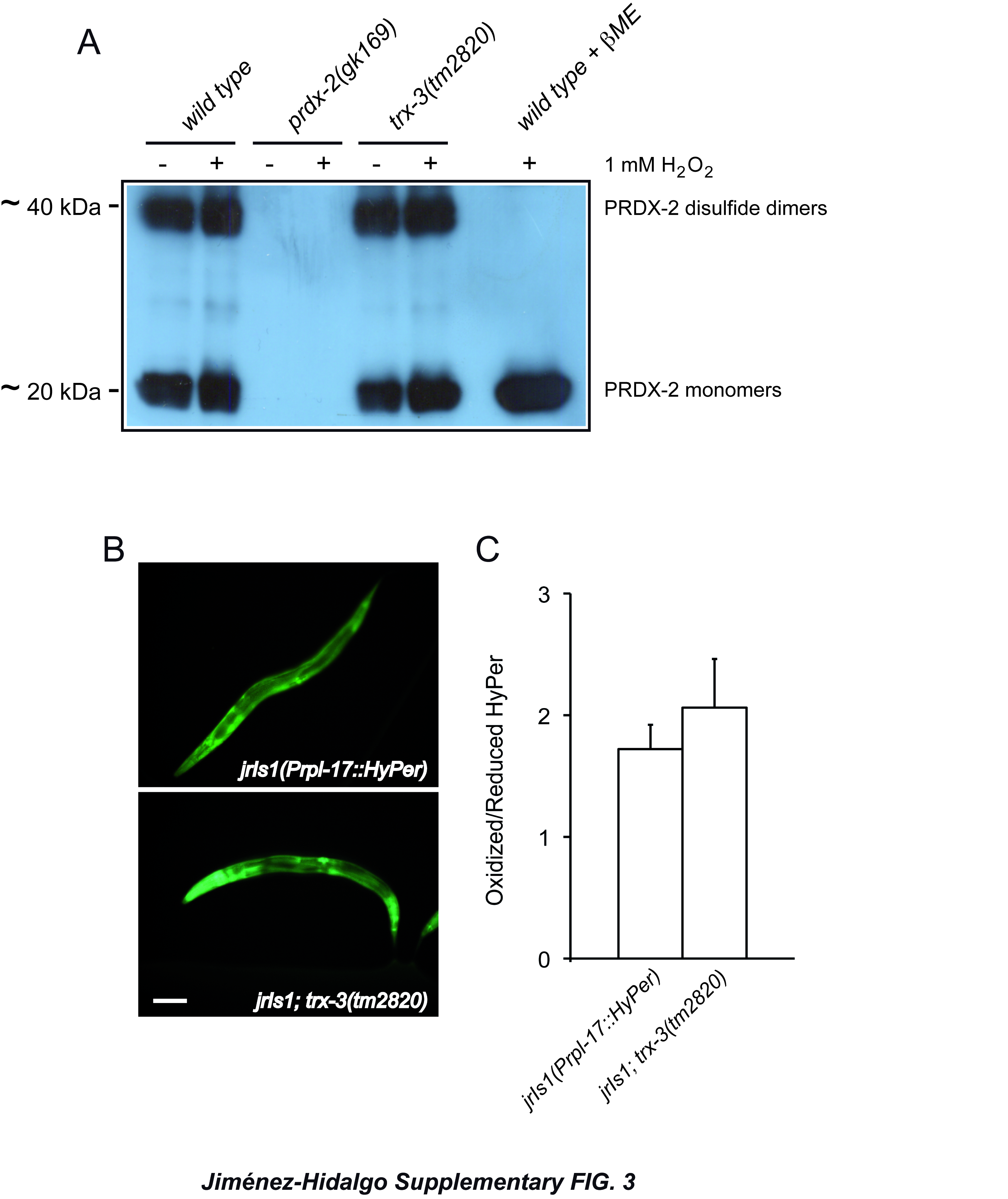

Supplement: Supplementary file 4 — Supplementary Material [file mmc4.zip › Supplementary Figure 3.tif]
